# Extensive lateral gene transfer between proto‐eukaryotes and *Heimdallarchaeia* suggests their close association during eukaryogenesis

**DOI:** 10.1002/mlf2.70030

**Published:** 2025-08-25

**Authors:** Patrick Forterre

**Affiliations:** ^1^ Institute Pasteur, Microbiology Department Paris France; ^2^ Institute for Integrative Biology of the Cell (I2BC), CEA, CNRS Univ. Paris‐Sud, Univ. Paris‐Saclay Gif‐sur‐Yvette Cedex France

**Keywords:** Asgard archaea, eukaryogenesis, eukaryotic specific proteins, *Hodarchaeales*, reverse gyrase

## Abstract

It has been proposed by Ettema and colleagues, in the two‐domain framework for the tree of life, that Eukarya emerged from *Heimdallarchaeia*, as sister group to *Hodarchaeales*. Looking at the individual trees of the protein markers used by these authors, I notice that Eukarya are only sister to *Hodarchaeales* or other *Heimdallarchaeia* in a minority of trees, whereas they are located far apart from these Asgard archaea in most other trees. Examination of single trees also reveals massive gene transfers from *Crenarchaeota* and/or *Korachaeota* to hyperthermophilic *Njordarchaeales*, explaining why their belonging to Asgard archaea is sometimes difficult to recover. Finally, I discuss several points raised by Ettema and colleagues, such as the phylogeny of Asgard archaea and the hyperthermophilic nature of their last common ancestor. The patchy localization of Eukarya in individual trees relative to *Hodarchaeales* and other *Heimdallarchaeia*, as well as the patchy distribution of eukaryotic signature proteins among Asgard archaea, is best explained by suggesting that multiple gene transfers take place between proto‐eukaryotes and Asgard archaea in both directions. This suggests that the co‐evolution of proto‐eukaryotes and Asgard archaea has played a major role in eukaryogenesis but also in shaping the physiology and diversification of Asgard archaea.

## INTRODUCTION

The discovery that a group of microorganisms superficially resembling bacteria (formerly *Archaebacteria*) was more closely related to Eukarya than to “classical” bacteria (formerly *Eubacteria*) was one of the most important discoveries in biology in the 20th century^1^, ^2^, ^3^, ^4^, ^5^, ^6^, ^7^, making the prokaryote/eukaryote classification, based on phenotypic observations, obsolete from an evolutionary point of view^6^, ^8^, ^9^. From that time, the evolutionary relationship between the so‐called three domains (3D) of life, Archaea, Bacteria, and Eukarya^10^, has been the subject of heated debates^11^. Whereas some authors have argued that Archaea and Eukarya are sister groups^10^, ^11^, ^12^, ^13^, ^14^, ^15^, ^16^, ^17^, ^18^, ^19^, sharing a common ancestor that was distinct from the two modern domains, others have argued that Eukarya (especially the nucleus) emerged from a subgroup of Archaea, following the endosymbiosis of one or two bacteria, depending on the model^20^, ^21^, ^22^, ^23^, ^24^, ^25^, ^26^, ^27^, ^28^, ^29^. Whereas Archaea, Bacteria, and Eukarya form three clearly distinct “domains” at the molecular level, independently of the proposed scenario, it is now usual to name the above two topologies either the 3D or the two domains (2D) trees, respectively^30^.

The 2D hypothesis has been strongly boosted by the discovery via metagenomic approaches of a new lineage of Archaea, the Asgard archaea, by Ettema and colleagues^23^, ^31^. In their first two publications describing this new superphylum, these authors obtained a 2D universal tree of life (uTol) in which Eukarya branch as sister group to one Asgard lineage^23^, ^31^. Moreover, the metagenome‐assembled genomes (MAGs) of Asgard archaea and the first two cultivated Asgard archaea^32^, ^33^ encode a bunch of proteins that are very similar to their eukaryotic homologues and are absent or rare in other Archaea. These so‐called ESPs (Eukaryotic Signature Proteins) were supposed to confirm the origin of Eukarya from Asgard archaea^31^, ^34^, ^35^, ^36^. In most current 2D scenarios for eukaryogenesis, the Asgard archaeon ancestor of Eukarya was radically transformed into a modern‐type eukaryote after it enclosed the alpha‐proteobacterium at the origin of mitochondria (early mitochondrial hypothesis)^24^, ^29^. In an alternative scenario (mitochondrial‐late hypothesis), the Asgard archaeon ancestor was engulfed by a bacterium at the origin of the eukaryotic membranes and became the eukaryotic nucleus, whereas mitochondria originated from a second endosymbiotic event^28^, ^37^.

In the first phylogenetic analysis performed by Ettema and colleagues, Eukarya were sister group to one of the three MAGs of Asgard archaea, called Loki 3^23^. In the following paper by the same group, when five lineages of Asgard archaea were recognized, Eukarya were still sister group to Loki 3, renamed *Heimdallarchaeote* LC_3^31^. In their more recent paper, in which the authors published phylogenetic trees based on the concatenation of proteins conserved between Archaea and Eukarya, Eukarya are deeply embedded within Asgard archaea, as members of the class *Heimdallarchaeia* ^36^. In this clade, they are sister group to one out of 15 lineages of Asgard archaea, corresponding to the order *Hodarchaeales*, which still includes the former Loki 3^36^.

In studying the individual phylogenies of the 36 universal proteins used as markers in the first publication of Ettema and colleagues (thereafter called the first Asgard paper)^23^, my colleagues and I detected a lot of pitfalls that explain why these authors recovered a 2D tree and obtained evidence in favor of a 3D tree^17^. For instance, we noticed that the elongation factor EF2 encoded by Loki 3 (*Hodarchaeales*) was much more similar to the eukaryotic EF2 than to the other Asgard EF2, and that removal of this single protein from the marker dataset was sufficient to eliminate the branching of Eukarya within Asgard archaea in the uTol, with Eukarya becoming sister group to *Korarchaeota* (fig. 4 in ref.^17^). We also reported that the 2D scenario was favored by three factors that converge to prevent the recovery of the short archaeal branch characteristic of the 3D tree^15^, ^17^. A first one is the overrepresentation of Archaea compared to Bacteria and Eukarya in the species dataset of the first Asgard paper, since it contained 84 archaea versus 10 bacteria and 10 eukaryotes^23^. Indeed, simulation analyses have shown that “oversampling of some group relative to other can influence the placement of other groups in the tree”^38^. Another factor that strongly favored 2D trees over 3D trees was the presence in the species dataset of fast‐evolving species, such as *Korarchaeota, Methanopyrales*, or DPANN (named from the first putative phylums detected in this putative superphylum, *Diapherotrites*, *Parvarchaea*, *Aenigmarchaea*, *Nanoarchaea*, and *Nanohaloarchaea*). Removing these fast‐evolving species from the dataset indeed increased the number of individual 3D trees from 1 to 11 (out of 36) as well as the recovery of the monophyly of the main archaeal groups known at that time: *Euryarchaeota, Crenarchaeota,* and *Thaumarchaeota* (tab. 1 in ref.^17^). A probable explanation is that fast‐evolving species tend to attract Archaea toward Bacteria, engulfing eukaryotic sequences in the process. The third factor favoring the recovery of 2D uTol was the abundance of small proteins carrying a low phylogenetic signal in the 36 markers' dataset^15^, ^17^. We observed a strong correlation between protein size and the recovery of either 2D or 3D uTol among the 36 proteins analyzed in the first Asgard paper; the longer the protein, the more likely the chance to recover robust 3D trees^17^. It is likely that small proteins cannot carry a signal corresponding to the short archaeal branch and, consequently, their concatenation cannot help recovery of this signal. Notably, there was also a good correlation between the length of the protein and the probability of recovering the monophyly of *Euryarchaeota, Crenarchaeota*, and *Thaumarchaeota*, indicating that long proteins produce more reliable trees (tab. S1 in ref.^17^).

Our paper analyzing the data from the first Asgard paper^17^ was either not cited in the subsequent literature or mostly criticized for our suggestion that some ESPs could have been due to contamination of Asgard archaea MAGs by environmental eukaryotic DNA. We made this suggestion because we observed two opposite topologies in the 36 trees of the first Asgard paper. We noticed that Eukarya were sister group to one, two, or three Asgard archaea in only 10 trees, whereas they were located far from them in the other 26 trees^17^. Moreover, the EF2 protein of the *Hodarchaeales* (formerly Loki 3) contained several eukaryotic‐like insertions that were absent in other Asgard archaea. A priori, this observation was best explained by the presence of contaminating eukaryotic DNA in the MAG of this Asgard archaeon. However, the discovery of a new member of *Hodarchaeales* (previously *Heimdallarchaeote* B3) makes this hypothesis unlikely^39^. Strikingly, *Hodarchaeales* branches as sister to Eukarya, but far from other lineages of Asgard archaea in the phylogenetic analyses of EF2 performed by the group of Ettema^39^. As an alternative hypothesis to the contamination scenario, my colleagues and I thus proposed that the ancestral EF2 protein of Asgard archaea was replaced in an ancestor of *Hodarchaeales* by the EF2 protein of a proto‐eukaryote^18^. This suggested the intriguing possibility that the phylogenies of some universal proteins could have also been affected by lateral gene transfer (LGT), possibly explaining why some of the 36 universal Asgard proteins studied by Ettema and colleagues were sister group to Eukarya in individual trees, whereas most of them were located far from Eukarya^17^.

Indeed, in reanalyzing the data published in 2021 by Li and colleagues^34^, my colleagues and I obtained strong evidence for LGT between proto‐eukaryotes and two lineages of Asgard archaea in the case of the universal protein Kae1/TsaD (a tRNA modification enzyme)^18^. In the tree obtained by Li and colleagues, Archaea were rooted within DPANN and the fast‐evolving *Methanopyrus kandleri* branch far from other *Euryarchaeota*, indicating that a strong bias was introduced in this tree by the presence of fast‐evolving species^18^. After removing DPANN and *M. kandleri* from the species dataset, we obtained a tree in which Eukarya branch as sister group to two families of *Heimdallarchaeia (Heimdallarchaeaceae* and *Kariarchaeaceae*), whereas all other Asgard lineages (including the other *Heimdallarchaeia*: *Hodarchaeales* and *Gerdarchaeales*) branch far from Eukarya^18^. This topology can be only parsimoniously explained by a single LGT between proto‐eukaryotes and a common ancestor of *Heimdallarchaeaceae* and *Kariarchaeaceae*. Notably, these two lineages indeed form a monophyletic clade in some Asgard phylogenies^34^, ^36^. Removing these two Asgard lineages from the dataset transformed the 2D tree into a 3D tree with all Asgard archaea nested within Archaea, far from Eukarya, indicating that LGT between proto‐eukaryotes and Asgard archaea is probably another misleading factor that has favored the 2D topology in recent analyses.

Two years ago, Ettema and colleagues published a new study in which they attempted to determine the position of Eukarya relative to Asgard archaea using an expanded dataset of Asgard lineages. They concluded that Eukarya belong to the class *Heimdallarchaeia*, as sister group to *Hodarchaeales* ^36^. This publication (thereafter called the *Hodar*/Eukarya paper) provided me with the opportunity to test more rigorously the LGT hypothesis based on a much larger dataset than the first Asgard paper, since it provides the possibility of analyzing the position of 14 Asgard archaea lineages in 113 individual trees. Moreover, since these trees include only Archaea and Eukarya, they are not affected by the long branch attraction effect produced in uTol by the very divergent bacterial proteins.

From the analysis of the 113 individual trees of the *Hodar*/Eukarya publication, I concluded here that the close relationships between Eukarya and *Heimdallarchaeia* observed by the authors do not reflect their parenthood but most likely testify for extensive LGT in both directions between proto‐eukaryotes and *Heimdallarchaeia*. This means that *Heimdallarchaeia* are not our ancestors, but that their study can nevertheless provide important information about eukaryogenesis. I also conclude from the analysis of the 113 trees that *Njordarchaeales* are bona fide Asgard archaeon, and that the difficulty in retrieving their correct position in some phylogenetic analyses is due to extensive LGT between these hyperthermophilic Asgard archaea and hyperthermophilic archaea related to *Korarchaeota* and *Crenarchaeota*.

I also discussed in this paper several aspects relevant to the *Hodar*/Eukarya publication, such as the nature of the Last Asgard Common Ancestor (LAsCA), the phylogeny of Asgard archaea, the rooting of the Asgard tree, and the patchy distribution of ESPs. Unlike the authors, I concluded that LAsCA did not encode reverse gyrase and was thus probably a mesophilic or a moderately thermophilic organism.

The various lineages of Asgard archaea were previously considered to form a phylum and the whole group was previously considered to form a superphylum^31^. This nomenclature has been challenged by Rinke and colleagues, who have proposed considering Asgard archaea as a single phylum, *Asgardarchaeota*, and the various Asgard lineages as inferior ranks (class, order, and families)^40^. More recently, Takai and colleagues proposed renaming this phylum *Promethearchaeota* ^41^. The global GTDB nomenclature raises serious issues, especially when the suffix archaea is replaced by the suffix bacteria for some major archaeal phylums^6^, ^42^, ^43^. Nevertheless, here, I will use this nomenclature in the case of Asgard archaea to be coherent with those used by Ettema and colleagues^36^, ^44^, ^45^. However, I will use the traditional nomenclature for major archaeal clades, such as *Crenarchaeota, Korarchaeota, Euryarchaeota*, or *Thaumarchaeota*, to be consistent with the historical nomenclature.

## DIFFERENT ROOTS FOR THE ARCHAEAL TREE

**Figure 1:**
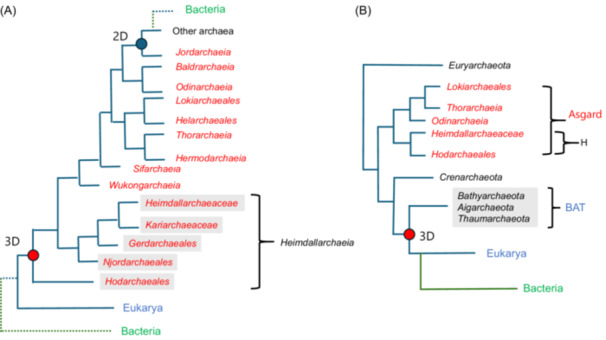
Comparison of the archaeal roots obtained by Ettema and colleagues in two domains (2D) or three domains (3D) frameworks with the root that we previously obtained in a 3D framework. (A) Schematic tree adapted from fig. 2 in ref.^36^, based on the concatenation of 36 non‐ribosomal proteins. The tree has been reorganized to show that in a 3D framework, the archaeal tree is rooted in *Hodarchaeales* (red circle) using Eukarya as the outgroup. The blue circle indicates the root of the archaeal tree in the 2D scenario. Bacteria, which are not include in the original tree, have been arbitrarily added with dotted green branches at their positions corresponding to either 2D or 3D trees. (B) Schematic tree adapted from the fig. S36 of ref.^17^, based on the concatenation of the two large RNA polymerase subunits. The red circle indicates the position of the root of the archaeal tree. H, *Heimdallarchaeia*; BAT, *Bathyarchaeota*, *Aigarchaeota* and *Thaumarchaeota*.

In their *Hodar*/Eukarya paper, Ettema and colleagues performed several phylogenetic analyses to determine the position of Eukarya relative to Asgard archaea, using two sets of proteins conserved in Archaea and Eukarya (but not necessarily present in Bacteria): a set of 56 ribosomal proteins (RP56) and a set of 57 non‐ribosomal proteins (NM57)^36^. They analyzed concatenations of these protein sequences based on two datasets containing either 64 or 175 Asgard MAGs (A‐64 and A‐175, respectively). They produced many unrooted trees obtained with or without fast‐evolving species (DPANN and *Korarchaeota*) with or without SR4 (state reduction 4) amino‐acid recoding and/or Fast‐Site Removal (FSR) treatments. The root of the archaeal tree turned out to be variable, depending on the analysis, Eukarya branching either as sister group to TACK (a putative superphylum including *Thaumarchaeota, Aigarchaeota, Crenarchaeota, Korarchaeota*, and their relatives), to a clade grouping TACK and *Njordarchaeales*, whose association with Asgard archaea was controversial^46^, to *Njordarchaeales* alone, to all Asgard archaea, or to *Hodarchaeales*. They performed statistical analyses of amino acid composition to disentangle the effect of temperature on the position of *Njordarchaeales* and *Korarchaeota* in their trees. They concluded that the NM57‐A‐175 dataset produces the best results and choose one of their analyses (performed without fast‐evolving species and with SR4 amino‐acid recoding) to illustrate their conclusion in the main text, that is, Eukarya are sister group to *Hodarchaeales* (fig. 2 in ref.^36^). In this figure, Eukarya and *Hodarchaeales* are sister groups because Ettema and colleagues have arbitrarily rooted the tree between Asgard archaea and other *archaea*, assuming that the 2D topology is already validated (blue circle in Figure 1A, adapted from fig. 2 in ref.^36^). In the obtained tree, *Hodarchaeales* and Eukarya are themselves sister to a clade grouping *Njordarchaeales, Gerdarchaeales, Heimdallarchaeaceae*, and *Kariarchaeaceae*. This clade (including Eukarya) corresponds to the class *Heimdallarchaeia*. Notably, in a stricto sensu cladistic nomenclature, if this topology is correct, it means that Eukarya is an order of *Heimdallarchaeia*.

In the absence of bacterial sequences, the trees obtained by Ettema and colleagues^36^ are not uTols and cannot provide new information about the 2D versus 3D debate. Surprisingly, this was unnoticed by one of the *Nature* reviewers, who wrote that “this paper will represent in my opinion the last “nail in the coffin” in support of a 2D tree of life”! Notably, if the 3D topology is correct^17^, ^18^, ^47^, the topology of their tree indicates that the archaeal tree should be rooted between *Hodarchaeales* and all other archaea (red circle in Figure 1A). This is in contradiction with previous analyses of 3D uTol that have suggested rooting the archaeal tree in the branch leading to the clade grouping *Thaumarchaeota* and relatives that we proposed naming the BAT clade (for *Bathyarchaeota*, *Aigarchaeota*, and *Thaumarchaeota*)^11^ (Figure 1B, adapted from fig. S33 in ref.^17^). This BAT root was first obtained before the discovery of Asgard archaea with a tree of archaeal ribosomal proteins including the *Thaumarchaeon Cenarchaeum symbiosum* and using eukaryotic sequences as the outgroup^48^. The BAT root was later recovered by Schleper, Brochier‐Armanet, and colleagues after the addition of more sequences from both mesophilic and thermophilic *Thaumarchaeota* and sequences from *Aigarchaeota* ^49^, ^50^.

Significantly, after the discovery of Asgard archaea, my colleagues and I obtained the BAT root with two robust Bayesian uTols based, for one of them, on the concatenation of the six universal proteins from the first Asgard paper that produced robust 3D uTol and, for the other, with the concatenation of the two large RNA polymerase subunits, using a balanced species dataset of 39 species for each domain^17^. Significantly, Embley and colleagues also obtained the BAT root with a 3D RNA polymerase uTol built using our dataset, but with a model supposed to be more efficient (fig. 6B in ref.^51^) (see the section “comparison with previous studies” in Supporting Information for more discussion about the RNA polymerase trees of Embley and colleagues). Notably, it has been shown later that the two RNA polymerase large subunits (the longest universal proteins) have the highest phylogenetic signal and outperformed a set of 41 conserved archaeal and bacterial markers in simulated phylogenetic reconstruction, despite having a shorter overall alignment length^38^. The two RNA polymerase large subunits thus appear to be essential to recover a proper uTol with the correct archaeal root and they are also most likely critical to obtain a proper archaeal phylogeny. Unfortunately, these two subunits are missing from the marker datasets used by Ettema and colleagues in which they recover the sisterhood of Eukarya and *Hodarchaeales* ^36^.

Interestingly, the BAT root of the archaeal tree is supported by the phylogeny of type I DNA topoisomerase (Topo IB). This enzyme is ubiquitous in BAT and in Eukarya but absent in all other archaea, with a few exceptions, including some Asgard archaea. Phylogenetic analyses of this enzyme produced 3D trees with a root in the BAT clade and Asgard archaea branching within BAT^52^, suggesting LGT from BAT to these Asgard archaea (see the Supporting Information and Figure S1 for more details).

I was interested in understanding why Ettema and colleagues recovered the root of the archaeal tree in the branch leading to *Hodarcheales* (in a 3D framework), whereas our previous phylogenies favored the BAT root. I have thus re‐examined the 113 trees published by these authors in their *Hodar*/Eukarya paper that can be recovered from the phylogenetic files called “A175_individual trees” of their figshare deposit. Although these trees include fast‐evolving species and variable number of archaeal sequences, their examination turned out to be useful to test the LGT hypothesis between Asgard archaea and proto‐eukaryotes.

### Analysis of the 113 trees suggests extensive gene transfers between Asgard archaea and proto‐eukaryotes

A major problem in the 113 individual trees that I could analyze is the extreme overrepresentation of Archaea versus Eukarya, since the trees in the A175 dataset are based on 14 eukaryotic sequences or fewer (see below) versus several hundred of archaeal sequences (usually between 330 and 400, the number of sequences that were used to build the trees being variable from one tree to another). The number of eukaryotic sequences is thus extremely low. Moreover, examination of the individual trees revealed that many of them contain fewer than 14 eukaryotic sequences and that three trees of the ribosomal proteins dataset and one tree of the non‐ribosomal proteins dataset do not contain a single eukaryotic species (arCOG0780, arCOG1885, arCOG4287, and MO32). These four proteins were thus included by mistake in the concatenation, and hereafter, I will name the RP56 and NM57 datasets RP53 and NM56 datasets, respectively. Several other trees only contain two or three eukaryotic sequences and should have also been discarded. Moreover, in a few trees, Eukarya are paraphyletic, suggesting LGT between Asgard archaea and modern Eukarya. Another similar major problem affecting the A175 dataset is that Asgard archaea are vastly overrepresented compared to other major groups of Archaea, since they usually represent around 50% of all archaeal sequences, with often more than 175 sequences. It is noteworthy that the final dataset of 331 genomes used by the authors for concatenation after streamlining was still highly unbalanced, with 175 Asgard archaea, 41 DPANN, 43 *Euryarchaeota,* and 72 TACK, versus 14 Eukarya.

These huge datasets and the massive overrepresentation of Archaea most likely explain why the branches leading from an archaeon or an archaeal clade to LECA (the Last Eukaryotic Common Ancestor) are very short in most of the 109 trees. This agrees with simulation experiments that have shown that increasing the number of sequences in the dataset led to a drastic reduction of internal branch length.^53^ On the other hand, the overrepresentation of Asgard archaea and the presence of fast‐evolving species probably explain why many nodes of these trees are unresolved with very short internal branches. Moreover, many clades contain a mixture of MAGs supposed to correspond to different archaeal groups and MAGs with similar annotation are often present in several unrelated clades, suggesting a high level of HGT and/or more likely mis annotation. It is noteworthy that some *Hodarchaeales* are still annotated as *Heimdallarchaea*, such as *Heimdallarchaeaceae*_LC_3 (formerly Loki 3), in the 113 trees.

It is important to verify that most individual trees show a reasonable degree of congruence before performing a concatenation^54^. However, one cannot distinguish congruent topology in comparing the 113 individual trees from each other. In the tree published by Ettema and colleagues after concatenation, Asgard archaea are monophyletic, except for Eukarya branching within Asgard archaea, as sister group to *Hodarchaeales*. However, this topology is never recovered among the 109 trees including Eukarya.

To evaluate the quality of the individual trees, I have been looking for the number of trees that recover the monophyly of Asgard archaea, with or without Eukarya branching within Asgard archaea. I found only six non‐ribosomal protein trees in which Asgard archaea are monophyletic (M038, M058, M060, M124, M198, and MA23), whereas they are paraphyletic in all ribosomal protein trees. In all these trees, Eukarya are branching apart from Asgard archaea. In four ribosomal protein trees and five non‐ribosomal protein trees, Asgard archaea are not monophyletic because *Njordarchaeales* are separated from other Asgard archaea, probably due to the replacement of the corresponding protein by the homologous protein from a hyperthermophilic archaeon related to *Korarchaeota* or *Crenarchaeota*.

Because of the very short branches of Eukarya in many trees and the very short branches that separate the various archaeal groups, I could not readily identify the sister group to Eukarya in 18 of the 53 ribosomal protein trees and in 14 of the 56 non‐ribosomal protein trees (Table 1). Eukarya branch in these “unresolved” trees sometimes at the crossroad of several archaeal clades separated by extremely short branches, sometimes nested within Asgard archaea and at the crossroad of several lineages, again separated by extremely short branches. In some cases, Eukarya are sister to clades containing MAGs from phylogenetically unrelated archaeal groups.

**Table 1:** Variability of eukaryotic position in the 113 trees of the *Hodar*/Eukarya paper^36^.

	RP56	NM57	Total
No Eukarya	3	1	4
Not resolved	18	14	32
Asgard archaea	23	27	50
***Heimdallarchaeia***	**15**	**19**	**34**
Other than Asgard archaea	12	15	27
***Hodarchaeales***	5	5	**10**
***Njordarchaeales***	4	6	**10**
***Heimdallarchaeaceae***	2	5	**7**
*Lokiarchaeales*	3	3	6
*Hermodarchaeia*	2	1	3
*Thorarchaeales*		3	3
***Gerdarchaeales***	1	1	**2**
***Njordarchaeales**/Korarchaeota*	1	1	**2**
*Jordarchaeia*	2		2
***Njordarchaeales/Gerdarchaeales***	1		**1**
***Kariararchaeacea**/Korarchaeota*		1	1
***Hodarchaeales/**Wukongarchaeia*	1		**1**
*Helarchaeales*		1	**1**
*Odinarchaeia*	1		1
BAT	5	3	8
*Crenarchaeota*	3	3	6
DPANN	2	4	6
*Euryarchaeota*	2	4	6
*Korarchaeaota*		1	1
	56	57	113

Each line indicates the number of trees in which Eukarya are sister group or more closely related to a particular lineage or to a particular clade of Archaea in individual trees of the ribosomal protein dataset (RP56) or in the new markers' dataset (NM57) of ref.^36^ composed of non‐ribosomal proteins. Eukaryotic sequences are missing in four trees. “Not resolved” indicates that one cannot distinguish easily by eye the sister lineage of Eukarya or that Eukarya are paraphyletic. The names of *Heimdallarchaeia* are in bold and in grey boxes. BAT, *Bathyarchaeota*, *Aigarchaeota*, and *Thaumarchaeota*; DPANN, *Diapherotrites*, *Parvarchaea*, *Aenigmarchaea*, *Nanoarchaea*, and *Nanohaloarchaea*.

When a sister clade to Eukarya can be identified with some confidence, they turned out to be sister to all possible archaeal groups, with nevertheless a predominance for Asgard archaea (Table 1). The numbers in Table 1 should be taken with a grain of salt because the position of Eukarya is highly dependent on the markers and species dataset. For instance, I notice that this position is often different in the A175 and A64 datasets. However, they show some interesting tendencies that are relevant for our understanding of the relationships between Eukarya and Asgard archaea. Overall, Eukarya are sister to a lineage of Asgard archaea in 50 resolved trees of the A175 dataset versus 27 for another archaeal group, indicating an overrepresentation of Asgard archaea that corresponds to about 50% of the species dataset. One can detect a strong enrichment of trees in which Eukarya are sister to *Heimdallarchaeia* (34 occurrences) and a slight enrichment of positions in which Eukarya are sister to *Lokiarchaeales* (6 occurrences). Eukarya are most often sister to *Hodarchaeales* or *Njordarchaeales* (10 occurences for *Hodarchaeales* and 10 for *Njordarchaeales*) followed by *Heimdallarchaeaceae* (7 occurrences). In only three cases, Eukarya are sister group to a clade grouping both *Hodarchaeales* and *Njordarchaeales*.

**Figure 2:**
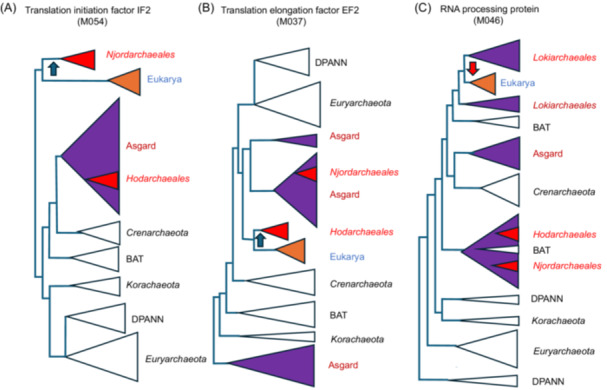
Schematic trees illustrating the variable position of Eukarya versus Asgard lineages in the individual trees obtained by Ettema and colleagues^36^. These schematic trees are adapted from the expanded trees published in the A175 tree file of the figshare deposit in ref.^36^. See Figure 1 for the complete names of the Asgard lineages. (A) Phylogenetic tree of the translation initiation factor IF2 suggesting a lateral gene transfer from a proto‐eukaryote to an ancestor of *Njordarchaeales* (blue arrow). (B) Phylogenetic tree of the translation elongation factor EF2 suggesting a lateral gene transfer from a proto‐eukaryote to an ancestor of *Hodarchaeales* (blue arrow). Note that *Njorarchaeales* and *Hodarchaeales* are far from each other in trees (A) and (B). (C) Phylogenetic tree of an RNA processing enzyme suggesting a lateral gene transfer from *Lokiarchaeales* to a proto‐eukaryote (red arrow). DPANN, *Diapherotrites*, *Parvarchaea*, *Aenigmarchaea*, *Nanoarchaea*, and *Nanohaloarchaea*.

**Figure 3:**
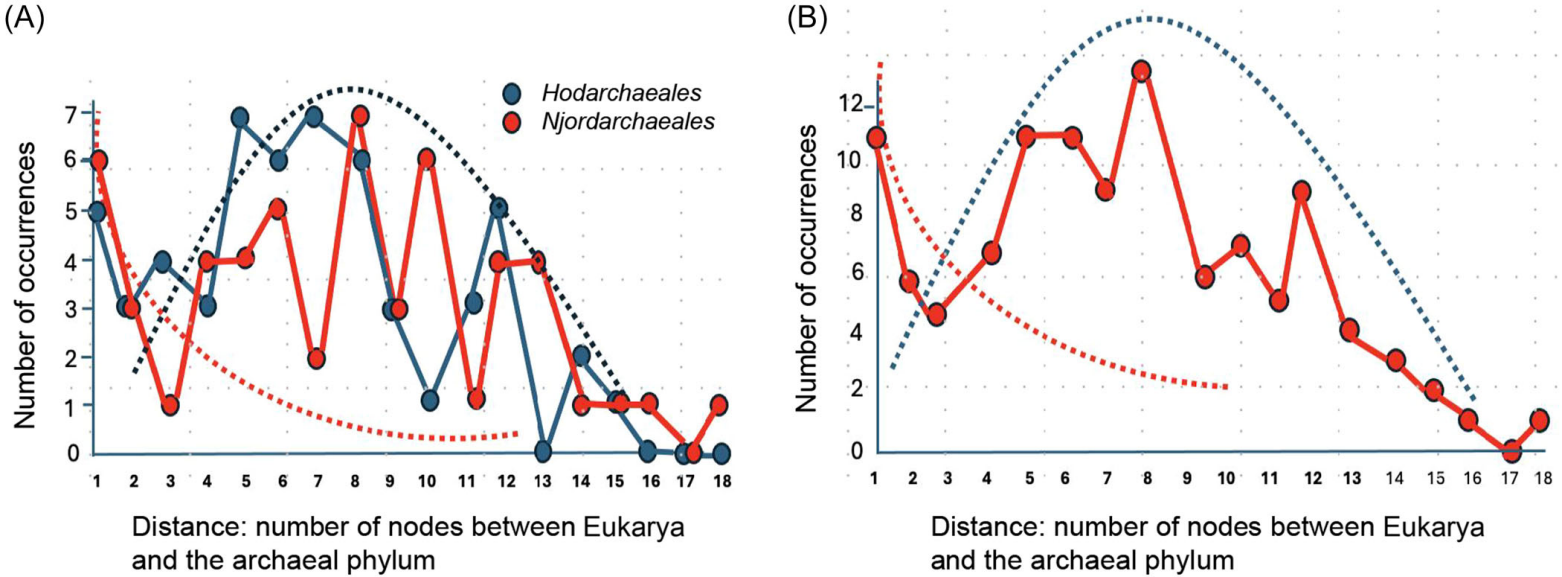
Evidence for two opposite signals in the position of Eukarya versus *Hodarchadeales* and *Njordarchaeales* in the trees that were concatenated by Ettema and colleagues^36^. (A) Number of individual trees (occurrences) as a function of the number of nodes separating Eukarya from either *Hodarchaeales* (blue curve) or *Njordarchaeales* (red curve) in these trees. Dotted curves correspond to schematic theoretical curves expected if a clade is sister group to Eukarya (red) or unrelated to Eukarya (blue). (B) Number of trees for *Hodarchaeale*s and *Njordarchaeales*. The results suggest the existence of two signals: one corresponding to trees affected by lateral gene transfer (LGT) between *Hodarchaeales* and *Njordarchaeales* and proto‐eukaryotes (red curve) and the other corresponding to the real phylogenetic position of the Eukarya versus *Hodarchaeales* or *Njordarchaeales* (blue curve).

The high number of trees in which *Heimdallarchaeia* are sister to Eukarya seems a priori to justify rooting the archaeal tree within this class of Asgard archaea. However, in contradiction with this conclusion, *Heimdallarchaeia* and *Heimdallarchaece*a branch far from Eukarya in the vast majority of the 109 trees. Moreover, in some trees in which either *Hodarchaeales, Njordarchaeales*, or another *Heimdallarchaeia* is sister to Eukarya, they are not always nested within other Asgard archaea, not even among other *Heimdallarchaeia*. For instance, in the tree of the translation initiation factor IF2 (M054), Eukarya and *Njordarchaeales* form an isolated clade far away from all other archaea, including other Asgard archaea that form a well‐defined monophyletic clade sister group to *Crenarchaeota* (Figure 2A). In the EF2 tree (M037), Eukarya are sister to a clade grouping 12 *Hodarchaeales* (including those annotated as *Heimdallarchaeceae* LC_3 and B3) with two other *Heimdallarchaeaceae* and one *Lokiarchaeales* (out of 67). This suggests that all EF2 in this clade belong in fact to *Hodarchaeales*, including the EF2 annotated as a lokiarchaeal protein. This clade branches between other Asgard archaea (including other *Heimdallarchaeia*) and *Euryarchaeota* (Figure 2B). This confirms the hypothesis that the ancestral EF2 protein of Asgard archaea was replaced in the *Hodarchaeales* by the EF2 of a proto‐eukaryote^18^. Notably, the EF2 protein of *Hodarchaeales* is now named “eukaryotic‐type EF2” in the ESP fig. 3 of the *Hodar*/Eukarya paper^36^. The authors thus have interpreted this EF2 as a specific *Hodarchaeales* ESP, supporting their proposed close relationships between *Hodarchaeale*s and Eukarya. It is noteworthy that this scenario would imply a dramatic acceleration of the EF2 evolution in the branch leading to *Hodarchaeale*s, followed by a long stasis during the diversification of Eukarya and *Hodarchaeales*.

### LGT from proto‐eukaryotes to Asgard archaea

The discrepancy between the position of heimdallarchaeial lineages in individual trees, sister to Eukarya in a few trees, but very distantly related from them in most others, suggests that *Heimdallarchaeia* are probably not closely related to Eukarya. The only logical explanation is that some trees in which Eukarya are closely related to a particular *Heimdallarchaeia* or to a subgroup of *Heimdallarchaeia* correspond to LGT between these Asgard archaea and proto‐eukaryotes as previously proposed for the universal proteins EF2 and TsaD/Kae1^18^. For instance, the replacement of the ancestral IF2 factor of an Asgard archaeon by an IF2 from a proto‐eukaryote seems the only possible logical explanation for the topology of the IF2 tree (Figure 2A).

To further test the LGT hypothesis in the case of *Hodarchaeales* and *Njordarchaeales*, I have examined the 56 trees including Eukarya of the non‐ribosomal protein dataset (NM57‐A175) counting visually the number of nodes between either *Hodarchaeales* or *Njordarchaeales* and Eukarya in each tree. This was challenging, since counting the number of nodes was difficult when branches separating clades are extremely short. However, since modifying the composition of the species dataset or the model used would have changed this number anyway, the numbers obtained here should not be taken at face value but as an indication for a given tendency.

By definition, Eukarya and their sister group are separated by one node. Table 2 shows the list of the 12 non‐ribosomal protein trees in which either *Hodarchaeales* or *Njordarchaeales* are separated by one node from Eukarya and one tree in which they form a clade separated from Eukarya by two nodes (Eukarya being sister group to *Korarchaeota* in this tree). One can see that *Njordarchaeales* are far from Eukarya (8 to 13 nodes) in four of the five trees in which *Hodarchaeales* are sister to Eukarya. Symmetrically, *Hodarchaeale*s are far from Eukarya (5 to 11 nodes) in the six trees in which *Njordarchaeales* are sister to Eukarya. In only three cases (M184, M050, and M127) *Hodarchaeales* and *Njordarchaeales* are both close to each other and to Eukarya. This suggests that a transfer has taken place between proto‐eukaryotes and a common ancestor of these two lineages of Asgard archaea. This supports the conclusion of Ettema, Spang, and colleagues that *Njordarchaeales* belong to the Asgard archaea^36^, ^56^, in contradiction with some previous phylogenies that suggested grouping *Njordarchaeale*s with *Korarchaeota* ^46^.

**Table 2:** Number of nodes between Eukarya and either *Hodarchaeales* or *Njordarchaeale*s in non‐ribosomal protein trees in which one of these two Asgard lineages is sister group to Eukarya.

Name in NM57	Protein name	*Hodar*	*Njord*
M184	DNA binding TFAR19‐related protein	1	2
M026	Serine/threonine protein kinase	1	8
M037	Elongation factor EF‐2	1	13
M191	Phosphopyruvate hydratase	1	13
M212	Pseudouridylate synthase subunit Tru B	1	12
M054	Translation initiation Factor IF‐2	8	1
M069	Methionine aminopeptidase	7	1
M078	Phe‐tRNA Synthetase alpha subunit	11	1
M080	2‐alkenyl reductase	5	1
M124	DNA Topoisomerase VI A subunit	5	1
M127	RecA/RadA recombinase	2	1
M050	Transcription factor TFIIB	2	2

The table lists the proteins from the NM57 marker dataset that are sister group to either *Hodarchaeales* (*Hodar*) or *Njordarchaeales* (*Njord)* in the trees of ref.^36^. The nomenclature and annotations are from ref.^55^. The numbers correspond to the number of nodes between Eukarya and either *Hodarchaeales* or *Njordarchaeales* in individual trees. The three yellow boxes correspond to trees in which both orders are close to Eukarya (separated by one or two nodes). The nine gray boxes correspond to trees in which one of the two orders of Asgard archaea is sister group to Eukarya, whereas the other is located far from Eukarya (from 5 to 13 nodes).

Figure 3A (blue curve) shows the number of individual trees among the 56 non‐ribosomal protein trees as a function of the number of nodes that separated *Hodarchaeales* from Eukarya. If the sisterhood between *Hodarchaeales* and Eukarya was real, one would have expected the most frequent trees (number of occurrence) being those in which *Hodarchaeales* and Eukarya are separated by one node, followed by a progressive decrease in the number of individual trees, as the distance between Eukarya and *Hodarchaeales* increases (theoretical curve with red dots). In contrast, if *Hodarchaeales* are unrelated to Eukarya, one would have expected a single Gaussian curve with very few trees corresponding to a single or two nodes and a maximum located at a rather high number of nodes (theoretical curve with blue dots). Instead, the curve obtained does not correspond to one of these two theoretical curves: starting from a rather high value corresponding to the number of trees in which *Hodarchaeales* are sister group to Eukarya (one node), followed by a small decrease for trees with two and three nodes and then by a progressive increase in the number of trees with four nodes or more, with a maximum of around seven nodes. It is noteworthy that the number of trees with five to eight nodes between Eukarya and *Hodarchaeales* was higher than the number of trees with one node.

The blue curve shows several peaks that can be explained by the variation in the number of internal branches from one tree to another and the difficulty in many cases in counting with confidence the exact number of nodes. To strengthen this first result, I thus performed a similar analysis with *Njordarchaeales* that are separated by two nodes in the phylogeny of Ettema and colleagues. I obtained a similar result (red curve). It is noteworthy that the number of trees with one node was higher than the number of trees with two nodes, whereas the number of trees with three nodes was very low. Combining the results obtained with both *Hodarchaeales* and *Njordarchaeales* (Figure 3B), the curves obtained can be more easily interpreted as bimodal curves corresponding to a combination of the red and blue dotted ones. One can conclude that most trees (above four nodes) illustrate the real position of *Hodarchaeales* and *Njordarchaeales*, far from Eukarya, whereas the most likely explanation for the excess of trees with one or two nodes (compared to the theoretical dotted blue curve) is that these trees testify for ancient LGT between proto‐eukaryotes and either *Hodarchaeales* and/or *Njordarchaeales*.

To summarize, the slight overrepresentation of Asgard archaea as sister group to Eukarya in the 109 trees can be explained by a combination of their overrepresentation in the species dataset (Eukarya branching randomly with a specific group according to the representation of this group in the dataset) and LGT between proto‐eukaryotes and Asgard archaea. These LGTs take place more frequently between Eukarya and *Heimdallarchaeia*, although some LGTs also involved other Asgard archaea, such as *Lokiarchaeales*.

**Figure 4:**
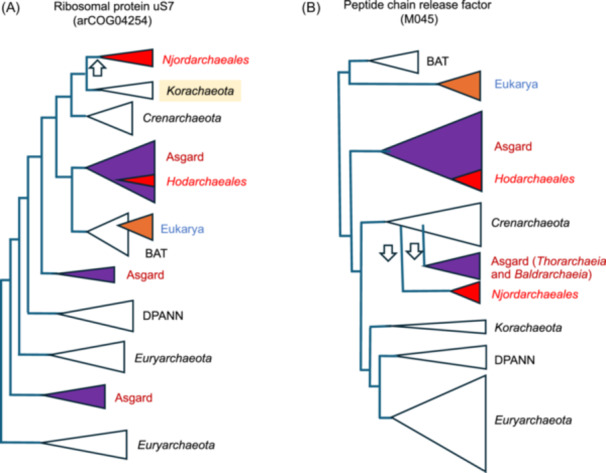
Schematic trees illustrating a LGT from *Korarchaeota* to *Njordarchaeales* and a possible rooting of the archaeal tree in the BAT clade (*Bathyarchaea, Aigarchaea,* and *Thaumarchaea*). These schematic trees are adapted from the trees published in the A175 trees file of the figshare deposit in ref.^36^. See Figure 1 for the complete names of the Asgard lineages. (A) Phylogenetic tree of the ribosomal protein uS7 strongly suggesting LGT of this protein from an ancestor of *Korarchaeot*a to *Njordarchaeales* (white arrow). (B) Phylogenetic tree of the peptide chain release factor, supporting the BAT root of the archaeal tree. Note that all major groups of Archaea are monophyletic in this tree, except Asgard archaea. The phylogeny suggests two independent LGTs (white arrows) from *Crenarchaeota* to *Njordarchaeale*s and from *Crenarchaeota* to *Thorarchaeia* and *Baldrarchaeia*.

LGTs between Asgard archaea and their partners in close association have probably been important throughout their co‐evolution. This can be observed in the case of *Njordarchaeales*. Whereas *Njordarchaeales* (hyperthermophiles) branch with other Asgard archaea in 51 of the 113 trees, they branch with *Korarchaeota* and/or *Crenarchaeota*, or with other non Asgard archaea, far from other Asgard archaea, in 38 and 18 trees, respectively, a surprisingly high proportion (Table 3). As a control, *Hodarchaeales* (mesophiles) branch with other Asgard archaea in 93 trees and close to *Korarchaeota* and/or *Crenarchaeota* or to other non Asgard archaea in only seven and five trees, respectively. Notably, the overrepresentation of trees in which *Njordarchaeales* are grouped with *Korarchaeota* and/or *Crenarchaeota* or with other non Asgard archaea is observed both with ribosomal and non‐ribosomal proteins (Table 3). The topologies of the 38 trees in which *Njordarchaeales* branch close to *Korarchaeota* and/or *Crenarchaeota* strongly suggest that the original Asgard proteins were replaced in *Njordarchaeales* by proteins from unrelated hyperthermophilic archaea living in close association with *Njordarchaeale*s in hot environments (see the example of the ribosomal protein uS7 in Figure 4A).

**Table 3:** Analysis suggesting the existence of extensive lateral gene transfer (LGT) between *Njordarchaeales* and other hyperthermophilic archaea that do not belong to Asgard archaea.

	*Njordarchaeales*		*Hodarchaeales*	
	Ribosomal RP56	Non‐ribosomal NM57	Ribosomal RP56	Non‐ribosomal NM57
Asgard archaea	23	28	50	43
*Kor/Cren*	20	18	3	4
Other archaea	10	8	3	2
Unresolved or missing	3 (a)	2 (b)	1	8 (c)

Number of trees in which either *Njordarchaeales* or *Hodarchaeales* (used as a control) branch either within or as sister group to other Asgard archaea (Asgard), or close (sister group or embedded) to either *Korarchaeota* or *Crenarchaeota* (*Kor/Cren*), or to other lineages of archaea. The numbers of trees in which *Njordarchaeales* are close to *Kor/Cren* are much higher than the corresponding numbers for *Hodarchaeales* for both ribosomal and non‐ribosomal proteins, suggesting extensive LGT between *Njordarchaeales* and other hyperthermophilic archaea. The fourth lane indicates the number of trees in which their position cannot be resolved with confidence (unresolved) or when they are missing: (a) absence of *Njordarchaeales* for arCOG04126, only one sequence of *Njordarchaeales* with *Crenarchaeota* for arCOG01885; (b) absence of *Njordarchaeales* for M047, *Njordarchaeales* paraphyletic for M037; and (c) absence of *Hodarchaeales* for protein M117.

It has been reported that *Njordarchaeales* are attracted toward *Korarchaeota* in phylogenetic analyses by similar amino acid composition^36^, ^56^. This bias toward amino acids preferentially used at high temperature was explained by the selection pressure of the environment. However, this does not fit with the correct grouping of *Njordarchaeales* with other Asgard archaea in many individual trees. The bias in amino acid composition could also be explained by the presence in *Njordarchaeales* of hyperthermophilic proteins recently recruited from relatives of *Korarchaeota* and/or *Crenarchaeota*.

### LGT from Asgard archaea to proto‐eukaryotes

If Asgard archaea and proto‐eukaryotes co‐evolved for a long time in the same biotopes, one should expect that LGT between these two groups has taken place in both directions. Transfer from a proto‐eukaryote to one or a few Asgard lineages can be deduced from the fact that other lineages of Asgard archaea are located far from Eukarya, as in the cases of the EF2 or the IF2 trees (Figure 2). On the other hand, in the case of a transfer from a particular Asgard lineage to proto‐eukaryotes, eukaryotic sequences should be embedded within those of Asgard archaea. This is often the case in resolved trees in which Eukarya are sister group to an Asgard lineage. However, it is not so easy to conclude in favor of LGT in these cases. The position of Eukarya within Asgard archaea could be simply due to the lack of resolution and/or the fact that Asgard archaea dominate the species dataset. Moreover, Asgard archaea are paraphyletic in most individual trees, confusing the interpretation even more. Proponents of the 2D model will argue that trees in which Eukarya are embedded within Asgard archaea support their favorite scenario (see the Supporting Information for examples in the recent literature). However, it seems difficult to use this argument to justify the unusual topology of most trees. This can be illustrated by the tree of a putative RNA processing enzyme (M046) in which eukaryotic sequences are deeply nested between various groups of *Lokiarchaeales* (Figure 2C), or the translation initiation factor IF1A (M052) in which eukaryotes are nested within *Helarchaeales*.

### The peptide chain release factor tree supports the BAT root

Notably, when Eukarya were not sister group to a lineage of Asgard archaea, the winner is the BAT clade, with eight occurrences, followed by the *Crenarchaeota*, the DPANN, and the *Euryarchaeota,* with six occurrences. One possibility is that this slight overrepresentation of BAT testifies for the rooting of the archaeal tree in the BAT branch, as we previously suggested. Interestingly, in the tree of the peptide chain release factor (M045), a rather large protein (360–400 amino acids), in which BAT and Eukarya are sister groups, Eukarya had a well‐defined long branch, and the consensus topology of the archaeal phylogeny is rather well conserved with BAT, *Crenarchaeota, Korarchaeota*, and *Euryarchaeota* being monophyletic (Figure 4B). Asgard archaea are paraphyletic, suggesting two independent LGT from *Crenarchaeota* to *Njordarchaeales* on one side and to *Baldrarchaeia* and *Thorarchaeia* on the other. The topology of the peptide chain release factor tree thus represents another significant data supporting the BAT root. This suggests again that more attention should be paid to this possible rooting by the scientific community. This is an especially important point, since, if the BAT root is correct, the GTDB proposed phylum *Thermoproteota* (grouping BAT with *Crenarchaeota* and a few other MAG‐defined archaeal lineages) is not a valid clade.

### LGT could explain the patchy distribution of eukaryotic signature proteins (ESPs) among lineages of Asgard archaea

With some exceptions, such as the Topo IB previously discussed, whose distribution and phylogeny support the BAT root (see the Supporting Information), the hypothesis of frequent LGT from proto‐eukaryotes best explains the patchy distribution of most ESPs^18^ than by suggesting that these ESPs are at the origin of their eukaryotic homologues. The problems raised by this patchy distribution, as well as by the extensive search for new ESPs using more and more sensitive methods are discussed in the supplementary material with several examples in the ancient and recent literature. Here, I focus on a few ESPs discussed by Ettema and colleagues in the *Hodar*/Eukarya paper. In this paper, the authors explored the expanded genomic diversity of Asgard archaea to look for new ESPs. They more than doubled the number of ESPs already known using various sensitive methods^36^. Their fig. 3 illustrates the extremely patchy distribution of ESPs among Asgard lineages. Only 18 out of 75 ESPs (the ubiquitous ESPs) are present in all or nearly all MAGs, the others being only present in a subset of Asgard lineages, sometimes (5 cases) in only one of them. Notably, in this figure, 21ESPs are missing in *Hodarchaeales*. This is, for instance, the case of the TRAPP components of the translocon complex whose compatibility with the eukaryotic machinery has been recently highlighted^57^. If *Hodarchaeales* are really sister group to Eukarya, this would imply that all the 21 missing ESPs were progressively introduced by LGT from various Asgard lineages into the sister lineage of *Hodarchaeales* at the origin of Eukarya and/or step by step during the evolution of proto‐eukaryotes^29^.

In an in‐depth analysis of archaeal ESCRT (Endosomal Sorting Complex Required for Transport) systems, Makarova and colleagues observed that *Hodarchaeales* lack many eukaryotic‐like components of the ESCRT systems and did not detect any synapomorphies between Eukarya and *Hodarchaeales* or other *Heimdallarchaeia* ^58^.

Ettema and colleagues pointed out the unique presence of a homologue of the eukaryotic DNA polymerase epsilon in *Hodarchaeales* as a possible synapomorphy supporting their grouping with Eukarya. In the phylogeny proposed by these authors^36^, this implies that epsilon‐like DNA polymerase of *Hodarchaeales* evolved from an archaeal DNA polymerase of the B family in the branch leading to *Hodarchaeales* after their divergence from *Njordarchaeales*. This seems very unlikely, since the epsilon‐like DNA polymerase detected in *Hodarchaeales* is very divergent from other DNA polymerases of the B family present in Asgard archaea^59^. Notably, various forms of B‐type DNA polymerases are present in all domains of life, and many of them being encoded by viruses and/or plasmids. It is thus more likely that the *Hodarchaeales* epsilon‐like DNA polymerase was introduced in this lineage by LGT from proto‐eukaryotes or from a mobile genetic element. Interestingly, on performing BLAST searches with the *Hodarchaeales* epsilon‐like DNA polymerases, I found a rather closely related homologue encoded by the Asgard archaeon *Candidatus* Lokiarchaeum ossiferum (NHK29699.1).

**Figure 5:**
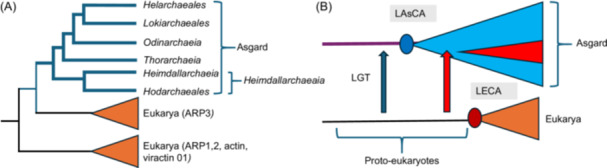
Possible scenario based on the LGT hypothesis explaining the existence of both ubiquitous (i.e., actin) and patchy distributed Eukaryotic Signature Proteins (ESPs) in Asgard archaea. See Figure 1 for the complete names of the Asgard lineages. (A) Schematic partial tree of asgardactin 01 (lokiactin) rooted with their closest neighbors in a global actin tree; adapted from the complete tree of actin and actin related proteins (ARPs) in ref.^60^. This tree suggests that an ancestor of ARP and actin was transferred to an ancestor of all modern Asgard archaea. (B) ESPs ubiquitous in Asgard archaea, such as asgardactin 01, should have been transferred from proto‐eukaryotes to Archaea before the emergence of the Last Asgard archaea Common Ancestor (LAsCA) (blue arrow), whereas ESPs with a patchy distribution in Asgard archaea were transferred during the diversification of Asgard archaea (red arrow and triangle). This indicates that proto‐eukaryotes were already present before the emergence of LAsCA, i.e., during the diversification of Archaea. LECA, Last Eukaryotic Common Ancestor.

In the case of ubiquitous ESPs, such as asgardactin 01 (Da Cunha et al.^60^), or Vps4^58^, one would expect, if *Hodarchaeales* and Eukarya were sister groups, these proteins to be paraphyletic in a phylogenetic analysis, with eukaryotic actin and Vps4 being sister group to their homologues in *Hodarchaeales*. In contrast, all Asgard actin 01 form monophyletic groups, with *Hodarchaeales* branching as sister group to other *Heimdallarchaeia*, far from their most closely related eukaryotic homologue, the actin‐related protein 3 (ARP3) (Supplementary fig. 2 in ref.^60^) (Figure 5A), whereas Asgard Vps4 form a monophyletic group that branches as sister group to Eukarya, far from other archaeal Vps4‐like proteins^58^. Notably, in the LGT hypothesis, the ubiquity of asgardactin 01 and VpS4 implies that these proteins were recruited from a proto‐eukaryote before the emergence of LAsCA (Figure 5B). This means that the first proto‐eukaryotes were already thriving before the diversification of Asgard archaea. On the other hand, since the transfers of some ESPs have been limited to subsets of Asgard lineages, or even to one, the proto‐eukaryotes were still thriving during the diversification of Asgard archaea (Figure 5B). This highlights the fact that proto‐eukaryotes co‐evolved with Archaea for a very long time before the emergence of the Last Eukaryotic Common Ancestor (LECA).

### The Asgard archaea species tree and its elusive root

**Figure 6:**
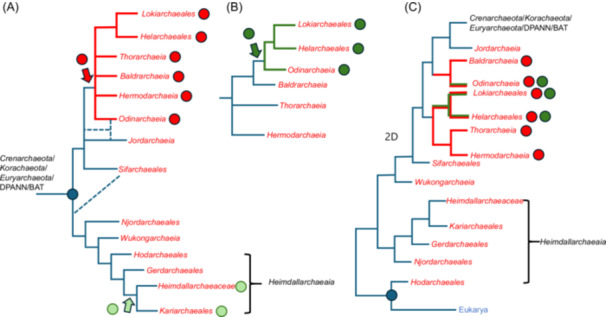
Insertions in two ribosomal proteins support an updated version of the consensus phylogeny for Asgard archaea. Alignments of the uS7 and uL16 insertions are illustrated in Figure S2. (A) Schematic tree adapted from fig. 2 in ref.^18^ (also called the consensus phylogeny in the text). Blue dotted lines indicate the alternative position of *Sifarchaeales* and *Odinarchaeia* in the tree of Spang and colleagues^56^ branches leading to lineages containing the uS7 insertio, are in red. (B) An improved version of part of this phylogeny with the monophyly of lineages containing the uL16 insertion branches leading to lineages containing the uL16 insertion are in green. (C) Schematic tree adapted from fig. 2 in the *Hodar*/Archaea paper^36^. Red and green circles indicate Asgard lineages containing the uS7 and uL16 insertions (Figure S2), respectively. Pale green circles indicate Asgard lineages containing single amino acid insertion in uL16. In (A) and (B), the Asgard lineages encoding the ribosomal proteins with the insertions are monophyletic, as indicated by the continuity of the red and green lines, respectively. In contrast, they are paraphyletic in (C), as indicated by the absence of continuity between the red lines and the green lines, respectively.

In their *Hodar*/Eukarya paper, Ettema and colleagues proposed a reconstruction of the nature of the LAsCA, based on a probabilistic gene tree species tree reconciliation approach supposed to reconstruct the ancestral gene content of LAsCA^36^. By definition, LAsCA is located at the root of the Asgard archaea tree. The root proposed by Ettema and colleagues, using *Euryarchaeota* and TACK as outgroups, is in the branch that separates *Jordarchaeia* from all other archaea^36^ (Figure 1A). This rooting is different from those obtained by several other authors^34^, ^35^, ^56^, ^61^, ^62^, ^63^, ^64^, ^65^. In our previous mLife review, we have proposed a “consensus” phylogeny of Asgard archaea by combining phylogenies published before 2022^18^ (Figure 6A). In this tree, the Asgard archaea are divided by the root into two groups: one grouping *Heimdallarchaeia* and *Wukongarchaeia*, and the other including all the other lineages. Spang and colleagues recently published a tree completely different from the tree of the *Hodar*/Eukarya paper, using TACK as the outgroup and a concatenation of 43 protein markers conserved in Archaea^56^. Notably, this phylogeny is rather similar to the consensus phylogeny that we proposed^18^, the two minor differences being the position of *Sifarchaeales* that branches on the other side of the root and the sisterhood of *Odinarchaeia* and *Jordarchaeia* (dotted lines in Figure 6A). I suspect that the phylogeny obtained by Spang and colleagues is better than those previously published by Ettema and colleagues because they have selected as markers for concatenation proteins producing individual trees in which the monophyly of major archaeal groups was correctly recovered^56^.

The rooting of the Asgard archaea tree between two main clusters (one of them including *Heimdallarchaeia*) is very similar to the rooting of the ubiquitous asgardactin 01 tree (Figure 5A)^60^. If this rooting is correct, the overlapping between the trees of Agard archaea and asgardactin 01 would strongly suggest that the transfer of actin from a proto‐eukaryote to Asgard archaea has taken place in the stem lineage of Asgard archaea, that is, before the emergence of LAsCA (Figure 5B).

An alternative rooting method has been proposed by Jüttner and Ferreira‐Cerca based on the organization of the 16S and 23S rRNA genes^66^. Whereas these two genes are linked into a complex operon in most Archaea, Bacteria, and Eukarya, they are unlinked in all Asgard archaea that the authors have analyzed for this character, except in *Odinarchaeia*. The unlinking of the 16S and 23S rRNA genes seems to be a rare and irreversible event observed in some fast‐evolving groups, such as DPANN. Moreover, there is no example of an evolutionary pathway that reverses the unlinking of rRNA genes^66^. This suggests that rRNA genes were still linked in LAsCA. In that case, the most parsimonious scenario is to place the root in the branch that separates Asgard with linked rRNA genes, such as *Odinarchaeia*, from Asgard with unlinked rRNA genes. However, it remains to be determined if the unlinking of rRNA genes has only taken place once during the evolution of Asgard archaea or if it has occurred several times independently in different lineages. It is important to screen more MAGs of Asgard archaea from different lineages for this character.

In the presence of contradictory phylogenetic analyses, the detection of significant insertions in protein sequence alignment can be a good source of information to validate or not the existence of some clades or to propose new ones. I recently identified in ribosomal proteins two large insertions present in a subset of Asgard archaea. The first one is an insertion (from 9 to 13 amino acids) shared by *Lokiarchaeales, Helarchaeales, Thorarchaeia, Baldarchaeia, Hermodarchaeia,* and *Odinarchaeia* (Figure S2). This insertion is also present in *Candidatus* Njordarchaeum guayamensis (MEX2689794.1). However, BLAST search with the region of the *Njordarchaeales* containing this insertion recovers a 100% similar sequence annotated as *Thermoproteota* (MCD6491924.1). We have seen that *Njordarchaeales* are sister group to *Korarchaeota*, far from other Asgard archaea in the uS7 phylogeny (arCOG04254) obtained by Ettema and colleagues (Figure 4A), suggesting that the ancestral uS7 protein from *Njordarchaeales* was replaced, via LGT, by the uS7 protein from a relative of *Korarchaeota* containing an insertion in the same region. The uS7 insertion was thus possibly not present in the ancestor of modern *Njordarchaeales*, before the LGT event. In that case, the Asgard archaea containing the uS7 insertions form a monophyletic group in the “consensus” phylogeny that we have proposed^18^ (Figure 6A). In the framework of this “consensus” phylogeny, the history of this insertion can indeed be explained by a single event (red arrow in Figure 6A) at the base of the clade grouping *Lokiarchaeales, Helarchaeales, Thorarchaeia, Baldarchaeia, Hermodarchaeia*, and *Odinarchaeia*. In contrast, the Asgard archaea containing the uS7 insertions do not form a monophyletic group in the tree published by Ettema and colleagues (Figure 5C) or in the tree published by Spang and colleagues. In the latter, *Odinarchaeia* that contain this insertion are sister group of *Jordarchaeia*, in which this insertion is absent^56^.

The presence of this insertion in a region of a universal protein otherwise fully conserved in Eukarya, in Bacteria, and in most Archaea is an indication of the high evolutionary rate of proteins from Asgard archaea. This is also exemplified by the variability of this insertion between the different lineages of Asgard archaea, even between different sequences of *Thorarchaeia*.

I further identified in the ribosomal protein uL16 a 6 to 9 amino acid insertion shared by a subgroup of the Asgard archaea cluster 1, including *Lokiarchaeales, Helarchaeales*, and *Odinarchaeia* (Figure S2). This insertion, which is also present in a highly conserved region of the protein, is missing in all other Archaea, except in DPANN, and in Eukarya. The size of this insertion is variable in *Lokiarchaeales*, again illustrating the rapid evolution of this protein sequence in Asgard archaea.

This insertion is missing in two MAGs of *Odinarchaeia* of the NCBI database (Figure S2), but BLAST search with these sequences did not retrieve other Asgard archaea uL16 proteins but uL16 from Archaea lacking this indel, such as “*Methalomethylicota”* or “*Culexarchaeota.”* This suggests contamination of these two *Odinarchaeia* MAGs by DNA from other Archaea lacking the insertion or LGT of uL16 from these archaea to these two *Odinarchaeia*.

A likely independent insertion of a single nucleotide in the same region, which probably occurred independently of the 6–9 insertion previously described, is present in *Kariarchaeales* and *Heimdallarchaeaceae*, in agreement with the sisterhood of these two lineages.

As in the case of the uS7 insertion, the distribution of this 6‐9 amino acid uL16 insertion can be explained by a single event in our “consensus” Asgard archaea phylogeny^18^ and further suggests that *Lokiarchaeales, Helarchaeales*, and *Odinarchaeia* form a monophyletic group nested within the clade defined by the uS7 insertion (Figure 6B). In contrast, Asgard archaea lineages containing this insertion do not form a monophyletic group in the trees of Ettema, Spang, and colleagues (Figure 6C).

### The nature of the LAsCA, hyperthermophile or not?

**Figure 7:**
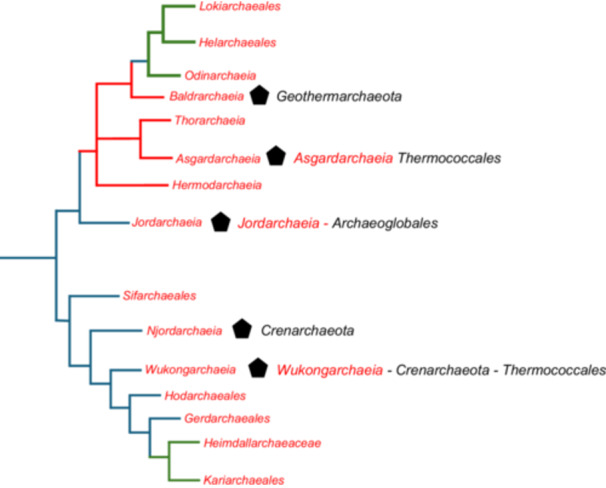
Distribution of reverse gyrase in an updated phylogenetic tree of Asgard archaea. The updated phylogenetic tree combining the trees of Figure 6A,B includes the position of *Asgardarchaeia* and the rooting obtained from the recent publication of Spang and colleagues^56^. The black pentagons indicate the presence of reverse gyrase in the corresponding Asgard lineage. The names at the right of the pentagons correspond to the organisms retrieved as first hits in BLAST search in the NCBI nr database, using the corresponding reverse gyrases as baits. Names in red correspond to first hits corresponding to Asgard archaea; all of them are recovered in the same lineages. BLAST searches with different reverse gyrases from *Njordarchaeia* (*Njordarchaeales* and *Panguiarchaeum*) retrived as first hits different subgroups of *Crenarchaeota.* The Asgard archaea reverse gyrases used in the analysis were first retrieved using the reverse gyrase of *Desulfurobacterium thermolithotrophum* DSM 11699 as bait.

Since the phylogeny of Asgard archaea and the root of their phylogenetic tree remain elusive, it seems premature to attempt reconstruction of LAsCA. I will thus not discuss here the proposal of Ettema and colleagues concerning the LAsCA metabolism but only focus on their claim that reverse gyrase was probably present in LAsCA, indicating that LAsCA lived at high temperature^36^. Reverse gyrase is indeed systematically present in all hyperthermophiles and some thermophiles, but not in mesophiles^67^, ^68^. Reverse gyrase is present in a rather high number of Asgard archaea lineages in both sides of the putative roots of the Asgard archaea tree^36^, ^41^, ^56^ (black pentagons in Figure 7). Searching for Asgard reverse gyrase in the NCBI database, I noticed that reverse gyrase is present in three MAGs of *Odinarchaeia*, but absent in all others, including well‐characterized MAGs such as *Candidatus* Odinarchaeum yellowstonii. Reverse gyrases from these three putative *Odinarchaeia* retrieved themselves as first hits in BLAST search, as well as reverse gyrases from *Candidatus* Asgardarchaeum abyssi and *Candidatus* Asgardarchaeum californiense. This suggests that reverse gyrase is missing in *Odinarchaeia* and that the three MAGs containing this enzyme and annotated as *Candidatus* Odinarchaeota archaeon should correspond to new MAGs of *Asgardarchaeia*.

Based on probabilistic gene tree species tree reconciliation approaches, Ettema and colleagues concluded in the summary and the main text of their *Hodar*/Eukarya paper that LAsCA was probably a thermophilic organism because it encoded reverse gyrase^36^. Using the same approach, Spang and colleagues also inferred with strong probability the presence of reverse gyrase in LAsCA, although based on a different phylogeny^56^. However, in contradiction with these statements, reverse gyrases from various Asgard lineages emerge at different positions embedded within reverse gyrases from different groups of hyperthermophilic archaea in the reverse gyrase phylogenies published by Ettema, Spang, and colleagues in three different publications (fig. 32 in ref.^36^, Supplementary fig. S1 in ref.^45^, and Supplementary fig. 33 in ref.^56^).

Notably, the authors are much more cautious in the texts of their supplementary material than in their summary and main texts. Ettema and colleagues wrote in their supplementary material that “the evolution of reverse gyrase is notoriously complex and that one cannot exclude that reverse gyrase was acquired several times independently at the base of Asgard archaea hyperthermophilic lineages”^36^, whereas Spang and colleagues wrote that “we cannot fully exclude the alternative scenario suggesting that reverse gyrase was acquired independently by distinct Asgard archaea rather than encoded by their common ancestor”^56^.

The names beside the reverse gyrases in Figure 7 indicate the first hits obtained in a BLAST search using these reverse gyrases as baits. In agreement with the reverse gyrase phylogenies published by Ettema, Spang, and colleagues, these BLAST searches only recover as first hits other Asgard archaea reverse gyrases of the same lineages (in red), followed by reverse gyrases from non Asgard hyperthermophilic archaea. In the case of the three *Njordarchaeales*, their reverse gyrases did not even retrieve each other's reverse gyrases but different reverse gyrase from *Thermoprotei*, suggesting independent gene transfers within this order. Similarly, the reverse gyrases of *Pangiarchaeum* branch far from those of various *Njordarchaeales* in the tree published by Spang and colleagues (Supplementary fig. 33 in ref.^56^), although both lineages have been grouped by these authors in the same class, *Njordarchaeia*, indicating another independent gene transfer within this class.

It has been shown that taxon of hyperthermophilic Asgard archaea, such as *Njordarchaeales* and *Panguiarchaeum,* are associated with taxa from *Crenarchaeota* and/or *Korarchaeota* in the metagenome community profile^56^. This cohabitation should have facilitated the transfer of reverse gyrase between these organisms, as well as the transfer of some of the markers that we detected in this study between *Njordarchaeales, Korarchaeota,* and/or *Crenarchaeota* (Table 3). LGT would have been even more facilitated if the Asgard archaea containing reverse gyrase were partners of other hyperthermophilic archaea in symbiotic (synthrophic) relationships. Interestingly, the origin of their reverse gyrase can provide an indication about the nature of the hosts of a particular lineage of thermophilic and/or hyperthermophilic Asgard archaea.

Thus, both phylogenetic analyses and BLAST searches indicate that reverse gyrases were several times independently transferred from various groups of hyperthermophilic archaea to Asgard archaea during their adaptation to life at high temperature, suggesting that LAsCA itself was a mesophile or possibly a moderate thermophile. Notably, this conclusion raises doubt about the methodology used by the authors to reconstruct the proteome of LAsCA. Since Eukarya (and most likely proto‐eukaryotes) cannot apparently thrive at temperature above 60°C^69^, it is likely that proto‐eukaryotes exchange exclusively some genes with mesophilic or moderately thermophilic Asgard archaea. Interestingly, the existence of LGT in both directions between proto‐eukaryotes (most likely mesophiles) and *Njordarchaeales* deduced from their positions in individual trees (Table 1) suggests that these LGT have taken place in the stem branch of *Njordarchaeales* before their adaptation to hyperthermophily, indicating a recent adaptation of these Asgard archaea to hot environments. This seems confirmed by the fact that reverse gyrases from three different *Njordarchaeales* have different origins.

In a parallel study, Li and colleagues attempted to reconstruct the evolution of the optimal growth temperature (OGT) during the evolution of Asgard archaea, using the elongation factor EF‐1A^70^ as a marker. They determined the optimal temperature for GTP binding of purified EF‐1A from modern Asgard archaeas and from recombinant EF‐1A based on sequences reconstructed at each node of an Asgard EF‐1A phylogeny. However, this phylogeny is different from those obtained with multiple protein markers or with the updated consensus phylogeny proposed here. In this EF‐1A phylogeny, the Asgard archaea tree is rooted between *Thorarchaeia* and all other Archaea. Li and colleagues obtained for LAsCA an OGT (65–75°C) higher than the OGT of Asgard archaea on both branches emerging from LAsCA. It is important to update this interesting approach using the consensus phylogeny described here and testing different possible roots. This again emphasizes the importance of determining the proper phylogeny and root of the Asgard archaea tree.

## DISCUSSION

In analyzing the individual trees published by Ettema and colleagues in the *Hodar*/Eukarya paper^36^, I notice several problems that raise doubts about the proposed position of Eukarya relative to the various lineages of Asgard archaea, the phylogeny of the Asgard archaea itself, and the reconstruction of their ancestor. These are the extreme lack of balance of Archaea versus Eukarya and Asgard archaea versus non Asgard archaea in their species dataset, the absence of important and reliable markers, such as the two large RNA polymerase subunits in the non‐ribosomal proteins dataset, and the use of methods such as SR4 recoding and FSR (Fast Site Removal) that can produce misleading results by removing valid phylogenetic signals. Some of these problems might have been noted by others, since a note from the *Nature* editor's now alerts the readers that “technical issues with the phylogenetic analysis of the NM57 dataset are being evaluated”^36^.

The main conclusion of the *Hodar*/Eukarya paper, based on the 2D scenario, was that Eukarya belong to *Heimdallarchaeia*, as sister group to *Hodarchaeales*, whereas in the framework of the 3D scenario, the results obtained suggested to root the archaeal tree between *Hodarchaeales* and all other Archaea. However, looking at the 113 individual trees of the *Hodar*/Eukarya paper, I notice that a close relationship between *Hodarchaeales* and Eukarya is only observed in a minority of tree (around 10%), whereas Eukarya and *Hodarchaeales* are located far apart from each other in all other trees. In fact, the analysis of the position of Eukarya in the 109 individual trees, including Eukarya, as well as the positions in these trees of *Hodarchaeales* and *Njordarchaeales* (the second Asgard archaea more closely related to Eukarya in the *Hodar*/Eukarya paper) both strongly suggest that *Hodarchaeales* are not closely related to Eukarya in evolutionary terms, but that *Hodarchaeale*s and other *Heimdallarchaeia* have exchanged many genes with proto‐eukaryotes, including ribosomal protein genes.

To test the possible effect of individual protein on the topology obtained after concatenation, Ettema and colleagues removed one protein from their non‐ribosomal protein marker dataset at a time and reported that this never changed the final topology^36^. This is not surprising, since the sisterhood between Eukarya and *Hodarchaeales* or *Njordarchaeales* was recovered five and six times in the non‐ribosomal protein datasets, respectively. Removal of a single marker thus cannot detect the bias introduced by the trees affected by LGT.

It is noteworthy that Takai and colleagues recently succeeded in isolating two members of the *Hodarchaeales* ^71^. Although it was previously speculated that Asgard archaea more closely related to Eukarya should have a more complex structure than the first isolated Asgard archaea (members of the *Lokiarchaeales*), microscopic observation of these *Hodarchaeales* revealed that both have simple internal cell structures with no membrane structures, in agreement with our conclusion that *Hodarchaeales* are not closely related to any eukaryotic ancestor. Finally, in a recent paper published by Dong and colleagues during the final revision step of this paper, the authors also conclude that Eukarya are not sister group to *Hodarchaeales* ^72^. Notably, four of the five non‐ribosomal proteins in which *Hodarchaeales* and Eukarya are sister groups in the dataset of Ettema and colleagues (Table 2) are absent in the 67 markers' dataset of Dong and colleagues, probably explaining the difference between the results obtained by these two groups. This again emphasizes the impact of marker selection in analyzing the position of Asgard archaea relative to Eukarya. Dong and colleagues concluded that Eukarya branch between the two major group of Asgard archaea, but they did not remove other markers affected by LGT between Asgard archaea and proto‐eukaryotes, probably explaining this result.

Despite the methodological problems linked to the *Hodar*/Eukarya paper, the trees published by Ettema and colleagues in figshare are extremely interesting. The overrepresentation of *Heimdallarchaeia*, especially *Hodarchaeales* and *Njordarchaeales*, as sister group to Eukarya (Table 1) suggests that preferential LGT have taken place between Eukarya and *Heimdallarchaeia* during eukaryogenesis, even if some LGT have also involved other groups of Asgard archaea, especially *Lokiarchaeales*. These observations strengthen our previous hypothesis that the signal supporting the close relationships between Asgard archaea and Eukarya in some uTol, as well as the patchy distribution of ESPs in various lineages of Asgard archaea, do not testify for an Asgard‐based 2D scenario but more likely to a long time co‐evolution between Asgard archaea and proto‐eukaryotes during eukaryogenesis, leading to extensive LGT in both directions between the two groups. The overrepresentation of *Heimdallarchaeia* among the partners of these transfers implies that *Heimdallarchaeia* were probably the most abundant Asgard archaea thriving with proto‐eukaryotes in the same biotopes.

The recent isolation of *Hodarchaeales* has revealed that these organisms are anaerobes that can only thrive in microaerobic environments in the presence of aerobic organisms^71^, suggesting that proteins involved in oxygen metabolism previously detected in their genomes^65^ were possibly involved in oxygen detoxification^65^, ^71^. This indicates the possibility that LGT has taken place between ancient *Heimdallarchaeia* and anaerobic or microaerobic proto‐eukaryotes. Besides *Heimdallarchaeia*, our observations suggest that *Lokiarchaeale*s were also present in the environments shared by proto‐eukaryotes and Asgard archaea.

Amazingly, considering the high number of proto‐eukaryotic proteins that have been transferred to Asgard archaea, these unique organisms can be viewed as Archaea that try to mimic Eukarya. The proto‐eukaryotes clearly should have also benefited from the input of genes recruited from Asgard archaea. There were mutual exchanges that influence both the evolution of proto‐eukaryotes and Asgard archaea.

The first four Asgard archaea that have been successfully cultivated are living in symbiosis with other microorganisms, mostly methanogenic archaea, interacting physically with their partners^32^, ^33^, ^71^. From comparative genomics analysis of Asgard archaea MAGs available at that time, Takai and colleagues suggested that LAsCA was a syntrophic organism, and that this lifestyle remains the rule during the evolution of Asgard archaea^32^. This would explain why these organisms are so difficult to cultivate and why only four of them have been successfully isolated 10 years after their discovery.

The close association between Asgard archaea and their partners, facilitating LGT between both, is also suggested by my analysis of *Njordarchaeales* position in the 113 trees. One can observe two opposite signals in this analysis: one (the majority) in which *Njordarchaeales* belong to Asgard archaea and one (a strong minority) in which they are closely related to *Crenarchaeota* or to *Korarchaeota*. I conclude from this observation that *Njordarchaeales* (hyperthermophiles) clearly belong to Asgard archaea and that their alternative positions in some analyses are most likely due to the extensive LGT that has taken place between them and hyperthermophilic archaea related to either *Crenarchaeota* or *Korarchaeota*. Notably, Dong and colleagues recently identified DNA from non Asgard hyperthermophiles in MAGs of *Njordarchaeales* and they also conclude that this presence, either due to contamination or LGT, explains the alternative positions of *Njordarchaeales* obtained by different authors^72^.

Organisms that are obligate symbionts of other organisms often show a high rate of protein evolution because they have been forced to adapt their physiology and structure to fit with this host–symbiont relationship. It is thus likely that proteins of Asgard archaea evolved more rapidly than average archaeal proteins. This fast‐evolving character is somehow exemplified by the two insertions detected here in subgroups of Asgard archaea, since these insertions are in regions of physiologically important proteins otherwise strictly conserved between Eukarya and most archaea. The fast‐evolving character of Asgard archaea would also explain why all of those that have been tested for this character, except *Odinarchaeia*, show unlinked rRNA genes. The unlinking of rRNA genes is indeed prevalent in fast‐evolving groups, such as the DPANN, and could be associated with syntropy/symbiosis^66^.

Usually, LGT involving informational proteins such as ribosomal proteins or other universal proteins is rare^73^. However, it has already been shown that such transfer can take place when the two partners physically interact. For instance, the exchange of ribosomal proteins between partners has been observed in studying the position of *Nanoarchaeum equitans* in the archaeal tree^74^. It was noticed that nine proteins of the large ribosome subunit from *N. equitans* branched with *Crenarchaeot*a, whereas most of the other branched at the base of the *Euryarchaeota*. This suggested that some ancestral DPANN ribosomal proteins were replaced in *N. equitans* by ribosomal proteins from relatives of their crenarchaeal host, *Ignicoccus hospitalis*. After the removal of proteins from the large ribosome subunit, my colleagues and I recovered *N. equitans* within *Euryarchaeota*. Notably, the same result has been obtained recently by Moreira and colleagues using an extensive set of protein markers^75^.

In this review, I observed that many exchanges of ribosomal proteins have probably taken place between *Njordarchaeales* and their putative hyperthermophilic partners. The analogy with the *N. equitans*/*I. hospitalis* system, in which the two partners physically interact, suggests that Asgard archaea and proto‐eukaryotes might have also physically interacted during eukaryogenesis. It would be important to study if such symbiosis still occurs in the modern biosphere between Asgard archaea and microbial eukaryotes. If this is the case, this would make more credible the existence of the ancient association between proto‐eukaryotes and Asgard archaea postulated here. Moreover, the study of modern partnerships between Eukarya and Asgard archaea could help infer the biology of these ancient associations and the mechanism of associated LGT.

## AUTHOR CONTRIBUTIONS

Patrick Forterre was responsible for the design of the paper, the data analyses and the writting.

## ETHICS STATEMENT

The publication discussed and analyzed in this review did not involve any experiments on animal or human.

## Supporting information

Supplementary material ital.
