# Supplementary material for: Extensive lateral gene transfer between proto‐eukaryotes and Heimdallarchaeia suggests their close association during eukaryogenesis
Source: mLife. 2025 Aug 25;4(4):345–62. doi: 10.1002/mlf2.70030 (PMC12395588; doi:10.1002/mlf2.70030)
Supplement: Supplementary file 1 — Supplementary material ital. [file MLF2-4-345-s001.docx]

**Extensive lateral gene transfer between proto-eukaryotes and *Heimdallarchaeia* suggests their close association during eukaryogenesis**

**Patrick Forterre**

**Supplementary material**

**Answer to criticisms of previous studies supporting the 3D scenario**

In the supplementary material of their Hodar/Eukarya paper (1), Ettema and colleagues discuss the results obtained previously by various groups compared to their own results. They claim that our critical analyses of their 2015 first Asgard paper in our PLoS Genetic papers in 2017 and 2018 (2, 3) were refuted by their own PLoS Genetic paper in 2018 (4). This presentation was misleading since our 2018 PLoS Genetic paper (3) was a rebuttal of the paper previously published the same year by Ettema and colleagues (4). One cannot refute a paper before its publication. Importantly, we tested in their rebuttal the possibility that the three domains (3D) topology of the RNA polymerase tree was due to the attraction of the eukaryotic branch by the long bacterial branch. We removed *Bacteria* from the species dataset and observed that the topology of the tree was not modified by the removal of *Bacteria*, with Asgard archaea still branching as sister group to *Euryarchaeota*, not to *Eukarya* (3).

Our 2017 PLoS Genetic paper is also often only cited to mention that we were wrong in suggesting that some MAGs of Asgard archaea were contaminated by eukaryotic DNA. Indeed, we suggested in this paper, but also in our 2018 rebuttal, that the eukaryotic-like features of the elongation factor 2 (EF2) present in *Hodarchaeales* (Loki 3) was probably a contaminant (2,3). However, Ettema and colleagues did not mention that we have recognized their mistake and suggested later that the EF2 from *Hodarchaeales* was more likely recruited from a proto-eukaryote by Lateral Gene Transfer (LGT) (5). Notably, when discussing the origin of Eukaryotic Signature Proteins (ESPs) in their PLoS Genetics 2018 paper, we already suggested that, beside possible contamination, the presence of ESPs in Asgard archaea “*could also be explained by* *ancient gene transfers with proto eukaryotes*” (5).

Ettema and colleagues also mentioned in the discussion of the Hodar/Archaea paper the 2020 publication by Embley and colleagues in the journal “*Nature Ecology and Evolution*” (6). In this publication, Embley and colleagues have focused on the 3D tree that we obtained in 2017 after removing both fast-evolving species and EF2 from the 36 proteins dataset of the first Asgard paper (2). Embley and colleagues succeeded to transform the 3D tree obtained with the remaining 35 proteins into a 2D tree using a different evolutionary model (6). This was not surprising, since the 35 tree was not made to settle the dispute between the 2D or 3D trees but to illustrate the effect of removal of a single protein, EF2, on the concatenation of the 36 proteins analyzed in the first Asgard paper. Accordingly, we used a rather simple model to save computing time, and we obtained a weakly supported 3D tree with Asgard archaea at the base of the Archaea (2). However, we obtained a strongly supported 3D tree when we concatenated the two large subunits of the RNA polymerase using a Bayesian method and a sophisticated model (2). Importantly, Embey and colleagues also recovered a 3D tree with their own evolutionary model when they use our dataset for their RNA polymerase phylogeny (6). They also recovered the root of the archaeal tree in the branch leading to the clade grouping *Bathyarchaeota, Aigarchaeota* and *Thaumarchaotea* (the *BAT* clade).

To obtain a 2D tree, Embley and colleagues needed to apply amino acid recoding the recoding SR4 procedure. However, in the tree thus obtained, the Asgard archaea are not sister to Eukarya but to a subgroup of *Euryarchaeota*, whereas Eukarya branch within paraphyletic *Crenarchaeota*, indicating that the SR4 recoding has produced an unreliable tree. The recovery of a 2D tree following amino-acid recoding can be easily explained by the fact that the internal branch leading to the monophyly of Archaea in the 3D topology disappears if the phylogenetic signal is reduced by decreasing the number of available characters (7). Indeed, simulation experiments have shown that fast evolving sites are highly informative for reconstruction of deep phylogenies and that the six states amino-acid recoding is not an effective strategy in the face of high saturation (7, 8). Hernandez and Ryan concluded from their analyses that “*the loss of information that accompanies six states recoding outweigh its benefit and therefore result in suboptimal phylogenetic reconstruction*” (7). Importantly, Rangel and Fournier noticed that including some fast-evolving sites in the analysis was essential to recover short internal branches in ancient phylogenies (8), this is precisely the case for the short branch testifying for the monophyly of *Archaea* in the 3D tree. In an independent paper, Embley and colleagues concluded from different simulations that recoding amino acids may either increase or decrease phylogenetic accuracy depending on the phylogenetic context (9). In the case of their RNA polymerase tree, the phylogenetic accuracy has been obviously dramatically decreased since the tree exhibits a staircase structure, with *Crenarchaeota* being paraphyletic (6). Surprisingly, despite recovering this unusual phylogeny, the authors observed that the model fit was substantially improved in their recoded tree, raising question about the meaning and validity of “best fitting” model.

Although the removal of fast-evolving sites is now extremely popular, one can notice that this strategy has produced in the past results that have not been confirmed later and/or are in contradiction with current knowledge about evolutionary relationships. For example, this strategy recovered the root of the universal tree in the eukaryotic branch (10), the rooting of the archaeal tree within *Euryarchaeota* (11), or else the sisterhood of *Nanohaloarchaea* and *Methanocellales* (12).

**The distribution and phylogeny of Topo IB support the *BAT* rooting and the 3D topology.**

If the root of the archaeal tree is located between the *BAT* clade and all other archaea, (2, 13), some ESPs present in this clade were possibly already present in the Last Asgard Common Ancestor (LAsCA) and later retained in *BAT* but lost in other archaeal lineages via a single evolutionary event. Some of them could have been later transferred from *BAT* to other archaeal lineages, including Asgard archaea. This is probably the case for the type I DNA topoisomerases of the B family (Topo IB) which is mentioned in the list of Asgard ESPs published by Ettema and colleagues in the Hodar/Eukarya paper (Figure 3 in reference 1). This enzyme is present in two homologous versions, a short one, which was first detected in some bacteria and in many viruses of the phylum *Nucleocytoviricota*, and a large one, which is ubiquitous in Eukarya and in the BAT clade (14). Topo IB was considered for a long time to be absent from Archaea. Its first archaeal representative was detected in *Thaumarchaeota* (15). The Topo IB from *Thaumarchaeota* branched as sister group to eukaryotic Topo IB in a tree rooted with the bacterial enzymes. This suggested that Topo IB was present in the last common ancestor of Archaea and Eukarya and later lost in the branch leading to *Crenarchaeota* and *Euryarchaeota*, supporting rooting the archaeal tree in the *BAT* clade (15). Topo IB was later detected in the genomes and MAGs of all other *BAT* members, but also in *Hadesarchaea* and in two lineages of Asgard archaea, *Gerdarchaeales* and *Thorarchaeia* (16, 17). In phylogenetic analyses, eukaryotic and archaeal Topo IB form two clearly distinct monophyletic groups and the Topo IB of *Hadesarchaea, Gerdarchaeales* and *Thorarchaei*a are nested within the *BAT* group, still in agreement with the BAT rooting (Supplementary figure S4 in reference 17) (Figure S1). This phylogeny suggests that Topo IB was transferred by LGT from *BAT* to *Thorarchaeia* and from *Thorarchaeia* to *Gerdarchaeales*.

**How to distinguish ESP from proteins already present in LAsCA.**

The case of Topo IB, most likely present in LAsCA, gives us clue about the possibility to distinguish protein that have been transferred from proto-eukaryotes to Asgard archaea from proteins already present in LAsCA. Strikingly, although archaeal Topo IB are much more similar to the eukaryotic ones than to the bacterial ones in sequence and structure, they are clearly distinct from their eukaryotic homologues, fitting with the definition of domain specific protein version proposed by Dieter Söll and colleagues (18). In the case of eukaryotic proteins, they wrote that “*a characteristic eukaryotic version of the molecule*” should be “*distinguishable from both the archaeal and the bacterial versions, but*” should be “*clearly of the archaeal genre*”. The possibility to identify an archaeal specific version of a protein could be a good criterion to distinguish ESPs that were already present in LAsCA from those introduced in Asgard archaea by LGT from proto-eukaryotes proteins. Notably, it is not possible to distinguish an archaeal version for many Asgard archaea specific ESPs because they are very similar to their eukaryotic homologues and/or they branch within eukaryotic proteins in phylogenetic analysis. This can be illustrated by the cases of Asgard actin and tubulin, two proteins supposed by the proponents of the 2D scenario to be at the origin of the eukaryotic cytosqueleton.

An Asgard tubulin was for a long time only present in *Odinarchaeia* (16, 5). This Asgard tubulin is very similar to its eukaryotic counterparts and clearly distinct from tubulin previously detected in Archaea, such as artubulin present in some *Thaumarchaeota*. Moreover, it is localized in a recent phylogenetic analysis between the various clades of tubulin present in Eukarya (19). Remarkably, a tubulin closely related to those of *Odinarchaeia* has been recently discovered in the *Lokiarchaeum* *Candidatus* Lokiarchaeum ossiferum (ID: [UYP44424.1](https://www.ncbi.nlm.nih.gov/protein/UYP44424.1?report=genbank&log$=protalign&blast_rank=6&RID=8V4ZRAF4016)) (20) and I detected new Asgard tubulins in two MAGs of *Heimdallarchaea* (ID: MDH5401501.1 and MDH5401500.1). The finding of tubulin in a single MAG of *Lokiarchaeales* or in only two MAGs of *Heimdallarchaea* among the hundreds of sequences present in the NCBI database can be best explained by LGT from proto-eukaryotes to Asgard archaea, followed by LGT between different Asgard isolates.

As in the case of Asgard tubulin, the five known types of actin present in Asgard archaea (asgardactins 01 to 05) are much more similar to the eukaryotic actin and to the eight known clades of eukaryotic actin related proteins (ARPs) than to actin homologues previously discovered in Archaea (crenactin) (20). In an in-depth phylogenetic analysis, we have obtained a tree rooted with crenactin, in which the various clades of asgardactins branch between the different clades of ARPs, whereas eukaryotic actin are nested with a group of actins encoded by giant viruses (viractins) that captured themselves their actin from proto-eukaryotes (Figure 5 of the main text) (20). Most of the ARP clades are widely distributed among eukaryotic groups, suggesting that these proteins were most likely already present in the Last Eukaryotic Common Ancestor (LECA). The simplest hypothesis explaining this topology implies several duplications of an ancestral genes that diverged from crenactin in the stem lineage of Eukarya, followed by several independent LGT from proto-eukaryotes toward different lineages of Agard archaea during eukaryogenesis. Notably, a relative of ARP sand asgardactin is present in *Bathyarchaeota*, indicating that LGT also occurred from proto-eukaryotes to this archaeal lineage (20).

**The search for ESPs should not be limited to Asgard archaea**

The discovery of actin in *Bathyarchaeota* (20) has shown that some ESPs previously believed to be specific of Asgard archaea can be present in other archaeal lineages, as it was already known for Topo IB, but also for some components of the ubiquitin or ESCRT systems. However, Ettema and colleagues did not indicate in the Figure 3 of their Hodar/Eukarya paper if some of the new ESPs described are also present in other groups of Archaea (1). This was also the case in the ESPs table published by Wang and colleagues (21). If the LGT hypothesis is correct, it is likely that some of the newly detected ESPs are present in other archaeal lineages that had also been in contact with proto-eukaryotes. It is also probable that some Asgard ESPs were recruited by LGT from other archaeal lineages, as in the case of Topo IB. I performed a few more BLAST searches with ESPs supposed to be specific for Asgard archaea and I retrieved homologous proteins in *Bathyarchaeota* in the case of tubulin (for instance ID: [MBT4422911.1](https://www.ncbi.nlm.nih.gov/protein/MBT4422911.1?report=genbank&log$=protalign&blast_rank=1&RID=8V5UVYTJ013)) or else several components of the translocon complex (the TRAPP alpha and beta subunits) that are absent from *Hodarchaeales*. For instance, looking for homologues of the *Lokiarchaeale*s proteins used by Robinson and colleagues in their analysis of the compatibility between the translocons of Asgard archaea and Eukarya, (22), I found homologues of the TRAPP alpha and beta subunits in MAGs of *Bathyarchaeota* (such as XHH07964.1 and MBD3187089.1, respectively) in *DPANN* and in *Bacteria*, whereas these two subunits were described as only present in *Eukarya* and Asgard archaea. Conversely, using as bait the beta-like TRAPP subunit of the coral *Pocillopora damicornis*, against Archaea, I recovered first hits in MAGs from *BAT* and Euryarchaeota. Moreover, doing BLAST with this same protein against the nr database without Eukarya, I first recovered many Bacteria before *Archaea*. It seems that a detailed and valid searches for all ESPs among the archaeal diversity remains to be done.

In a more recent work, Ettema and colleagues have made a lot of efforts to look for new ESPs in Asgard archaea, using more sensitive sequence similarity detection algorithms and/or de novo protein structure modeling continuously expending the number of ESPs (1, 23). Unfortunately, as in the case of previously detected ESPs, they did not mention if they have look for the presence of these ESPs with similar methods in other archaeal groups. Moreover, in contrast to previously described ESPs, some of these new putative ESPs are very divergent from their eukaryotic homologues, making difficult the reconstruction of their evolutionary history.

**Recent data from the literature suggest more examples of lateral gene transfer between Asgard archaea and proto-eukaryotes.**

Several possible examples of gene transfer between Asgard archaea and proto-eukaryotes can be found in the recent literature. In a paper describing the distribution of Argonaute (Argo) proteins in *Archaea* and *Bacteria*, the authors detected an Argo protein (HrAgo1) in the MAG of the *Lokiarchaeon* *Candidatus* Harpocratesius repetitus that branches between eukaryotic AGO and PIWI, far from all other archaeal and bacterial AGO proteins, the latter branching at all possible positions in Archaea and Bacteria (Figure 1 in reference 24). In agreement with the 2D paradigm, they conclude that HrAgo1 could be the ancestor of all modern eukaryotic AGO proteins. However, the presence of HrAgo1 being restricted to *Candidatus* Harpocratesius repetitus, it is much more likely that it was transferred from a proto-eukaryotes to an ancestor of *Candidatus* Harpocratesius repetitus. Another recent example can be found in the tree of the antiviral proteins viperine and Argonaute (25). In the viperine tree, eukaryotic proteins, closely related to some cyanobacterial viperin, are nested within Asgard archaea, themselves nested within bacterial viperins, whereas in the Argonaute phylogeny, Eukarya are sister group to a subgroup of Asgard archaea, nested within a mixture of archaeal, bacterial and Argonautes from other lineages of Asgard archaea.

In an analysis of the enzymes involved in central carbon metabolism, Spang and colleagues produced several phylogenies strongly suggesting LGT between proto-eukaryotes and Asgard archaea (26). For example, in the tree of ADP-dependent glucokinase (ADPGK), Eukarya are sister group to two *Heimdallarchaeia* and two other *Heimdallarchaeia* are sister group to *Bacteria*, this enzyme being apparently absent from all other Asgard archaea. The grouping of Eukarya with subgroups of *Heimdallarchaeia* is also observed in the trees of 2,3-bisphosphoglycerate-independent phosphoglycerate mutase (GPMI) and enolase (ENO). In the enolase tree, the clade formed by Eukarya and *Heimdallarchaeia* is nested within *DPANN* and three other *Heimdallarchaeia*, whereas another group of eukaryotic ENO is nested within other archaea as sister to *Thermoplasmatot*a. Interestingly, *Eukarya* are sister to three *Lokiarchaeales*, far from other Asgard archaeas, in the GPMI tree, and from six *Lokiarchaeales*, again far from other Asgard archaea in the tree of the acetyl-Coa synthetase (ACDA). Finally, Eukarya are nested within *Lokiarchaeales* in the tree of ribose 5-phosphate isomerase (RPIA), as in the RNA processing enzyme of the 113 trees dataset, and sister to *Lokiarchaeia*, nested within Asgard archaea, in the tree of pyruvate kinase (PK). In agreement with the current 2D paradigm, the authors interpret their result as indication that these eukaryotic enzymes originated from the Asgard archaea ancestor, whereas a network of LGT between some lineages of archaea and proto-eukaryotes seems much more likely.

Other recent examples of transfers from Asgard archaea to proto-eukaryotes can be found in the phylogenies of three enzymes of the mevalonate pathway used to synthesize isoprenoids: mevalonate kinase (MVK), phosphomevalonate kinase (PMK) and a diphosphomevalonate decarboxylase (DMD) (26). In the MVK tree, Eukarya are sister group to a subgroup of *Heimdallarchaeia*, far from *Hodarchaeales* and other Asgard archaea (26). In the phylogeny of PMK and DMD, Eukarya are not sister to some lineage of Asgard archaea but to *Sulfolobales*, indicating in that case a possible transfer from *Sulfolobale*s to proto-eukaryotes (26).

Finally, the early LGT hypothesis from proto-eukaryotes to Asgard archaea before the emergence of LAsCA could explain recent phylogenies in which ubiquitous ESPs form clades sister group to *Eukarya*, as in the case of the Vps4 phylogeny (28) whereas transfer from specific Asgard lineage to proto-eukaryotes could explain phylogenies in which some eukaryotic proteins branch within Archaea, as in the case of the A subunit of the DNA topoisomerase VI (Topo VI) that branches from *Heimdallarchaeia* (29). The transfer of Topo VI A subunit from Asgard archaea to proto-eukaryotes would have been an important contribution of Asgard archaea to eukaryogenesis since the Topo VI was later recruited in *Eukarya* to provide the protein complex that cleaves the chromosomes during meiotic recombination (30, 31).

In all these recent publications, none of the authors even mention the possibility of LGT from proto-eukaryotes to explain their results, testifying for the powerful impact of the 2D paradigm in the present scientific community working on Archaea. In contrast, the discovery of a few bacterial actin and tubulin branching within eukaryotic sequences (32-37) was readily explained by the authors as a case of LGT between proto-eukaryotes and bacteria.

**Specific amino-acid insertions that are present in the sequences of ribosomal proteins uS7 and uL16 of Asgard archaea can help to identify the correct topology of the Asgard tree.**

Homologous insertions in the uS7 and uL16 ribosomal proteins are present in some lineages of Asgard archaea. Some of these insertions are illustrated in Figure S2. These insertions are present in all members of each indicated lineages, except for the uL16 insertion that is only present in a subset of MAGs annotated as *Odinarchaeia* (the figure shows two examples with or without the insertion). These long homologous insertions cannot have been introduced independently by chance in different lineages of Asgard archaea. Accordingly, they are helpful to evaluate Asgard archaea phylogenies because they can be used as synapomorphies to define clades, based on the parsimonious principle. The presence of these insertions in otherwise highly conserved regions of ribosomal proteins and their variability between different lineages of Asgard archaea suggest that proteins encoded by Asgard archaea evolve faster than the average archaeal proteins.

**References**

1. Eme L, Tamarit D, Caceres EF, Stairs CW, De Anda V, Schön ME, et al. Inference and reconstruction of the heimdallarchaeial ancestry of eukaryotes*. Nature*. 2023; 618:992-9.
2. Da Cunha V, Gaia M, Gadelle D, Nasir A, Forterre P. Lokiarchaea are close relatives of Euryarchaeota, not bridging the gap between prokaryotes and eukaryotes. *PLoS Genet*. 2017; 13(6):e1006810. doi: 10.1371/journal.pgen.1006810.
3. Da Cunha V, Gaia M, Nasir A, Forterre P. Asgard archaea do not close the debate about the universal tree of life topology. *PLoS Genet*. 2018; 14(3):e1007215. doi: 10.1371/journal.pgen.1007215.
4. Spang A, Eme L, Saw JH, Caceres EF, Zaremba-Niedzwiedzka K, et al. Asgard archaea are the closest prokaryotic relatives of eukaryotes. *PLoS Genet*. 2018; 14: e1007080.
5. Da Cunha V, Gaïa M, Forterre P. The expanding Asgard archaea and their elusive relationships with Eukarya. *mLife*. 2022; 1:3-12.
6. Williams TA, Cox CJ, Foster PG, Szöllősi GJ, Embley TM. Phylogenomics provides robust support for a two-domains tree of life. *Nat Ecol Evol*. 2020; 4: 138–47.
7. Hernandez AM, Ryan JF. Six-State Amino Acid Recoding is not an Effective Strategy to Offset Compositional Heterogeneity and Saturation in Phylogenetic Analyses. *Syst Biol*. 2021; 70: 1200-12.
8. Rangel LT, Fournier GP. Fast-evolving alignment sites are highly informative for reconstructions of deep Tree of Life phylogenies. *Microorganisms*. 2023; 11:2499. doi: 10.3390/microorganisms11102499.
9. Foster PG, Schrempf D, Szöllősi GJ, Williams TA, Cox CJ, Embley TM. Recoding Amino Acids to a Reduced Alphabet may Increase or Decrease Phylogenetic Accuracy. *Syst Biol*. 2023; 72:723-37.
10. Brinkmann H, Philippe H. Archaea sister group of Bacteria? Indications from tree reconstruction artifacts in ancient phylogenies. *Mol Biol Evol*. 1999; 16:817-25.
11. Raymann K, Brochier-Armanet C, Gribaldo S. The two-domain tree of life is linked to a new root for the Archaea. *Proc Natl Acad Sci U S* *A*. 2015; 112:6670-5.
12. Aouad M, Taib N, Oudart A, Lecocq M, Gouy M, Brochier-Armanet C Extreme halophilic archaea derive from two distinct methanogen Class II lineages*. Mol Phyogenetl Evol.* 2018;127:46-54.
13. Eme L, Reigstad LJ, Spang A, Lanzén A, Weinmaier T, Rattei T, Schleper C, Brochier-Armanet C. Metagenomics of Kamchatkan hot spring filaments reveal two new major (hyper)thermophilic lineages related to Thaumarchaeota. *Res Micro*biol. 2013 ; 164:425-38.
14. Takahashi DT, Gadelle D, Agama K, Kiselev E, Zhang H, Yab E, et al. Topoisomerase I (TOP1) dynamics: conformational transition from open to closed states. *Nat Commun*. 2022; 13(1):59. doi: 10.1038/s41467-021-27686-7.
15. Brochier-Armanet C, Gribaldo S, Forterre P. A DNA topoisomerase IB in Thaumarchaeota testifies for the presence of this enzyme in the last common ancestor of Archaea and Eukarya. *Biol Direct*. 2008; 3:54. doi: 10.1186/1745-6150-3-54.
16. Zaremba-Niedzwiedzka K, Caceres EF, Saw JH, Bäckström Di, Juzokaite L, Vancaester E, et al. Asgard archaea illuminate the origin of eukaryotic cellular complexity. *Nature*. 2017; 541: 353–8.
17. Cai M, Liu Y, Yin X, Zhou Z, Friedrich MW, Richter-Heitmann T, et al. Diverse Asgard archaea including the novel phylum Gerdarchaeota participate in organic matter degradation. *Sci China Life Sci*. 2020; 63:886-97.
18. Woese CR, Olsen GJ, Ibba M, Söll D. Aminoacyl-tRNA synthetases, the genetic code, and the evolutionary process. *Microbiol Mol Biol Rev*. 2000; 64:202-36.
19. Santana-Molina C, Del Saz-Navarro D, Devos DP. Early origin and evolution of the FtsZ/tubulin protein family. *Front Microbiol*. 2023 Jan 10;13:1100249. doi: 10.3389/fmicb.2022.1100249.
20. Da Cunha V, Gaia M, Ogata H, Jaillon O, Delmont TO, Forterre P. Giant viruses encode novel types of actins possibly related to the origin of eukaryotic actin: the viractins. *Mol Biol Evol*. 2022*,* 39(2):msac022. doi: 10.1093/molbev/msac022.
21. Xie R, Wang Y, Huang D, Hou J, Li L, Hu H, et al. Expanding Asgard archaea

members in the domain of Archaea sheds new light on the origin of eukaryotes. *Sci China Life Sci*. 2021 ; 65:818-29.

1. Carilo I, Senju Y, Yokoyama T, Robinson RC. Intercompatibility of eukaryotic and Asgard archaea ribosome-translocon machineries. *J Biol C*hem. 2024; 300(9):107673. doi: 10.1016/j.jbc.2024.107673.
2. Köstlbacher S, van Hooff JJE, Panagiotou K, Tamarit D, De Anda V, Appler KE, et al. Structure-based inference of eukaryotic complexity in Asgard archaea. *bioRxi*v. 2024; doi.org/10.1101/2024.07.03.601958.
3. Bastiaanssen C, Ugarte PB, Kim K, Feng Y, Finocchio G, Anzelon TA, et al. RNA-guided RNA silencing by an Asgard archaeal Argonaute. *Nat Commun*. 2024;15, doi.org/10.1038/s41467-024-49452-1.
4. Leäo P, Little ME, Appler KE, Sahaya D, Aguilar-Pine E, Currie K, et al. Asgard archaea defense systems and their roles in the origin of eukaryotic immunity. *Nat Commun*. 2024; 15(1):6386. doi: 10.1038/s41467-024-50195-2.
5. Santana-Molina C, Williams TA, Snel B, Spang A, Chimeric Origins and Dynamic Evolution of Central Carbon Metabolism in Eukaryotes. 2024; *bioRxiv*. 2024; https://doi.org/10.1101/2024.05.29.596406.
6. Zhu P, Hou J, Xiong Y, Xie R, Wang Y, Wang F. Expanded Archaeal Genomes Shed New Light on the Evolution of Isoprenoid Biosynthesis. *Microorganism*s. 2024; 12(4):707. doi: 10.3390/microorganisms12040707.
7. Makarova KS, Toliasson V, Wolf YI, Lu Z, Liu Y, Zhang S et al., Diversity, origin, and evolution of the ESCRT systems. *mBio*, 5(3):e0033524. doi: 10.1128/mbio.00335-10.1128/mbio.00335-24
8. Huang, WC, Probst M, Hua ZS, Szánthó LL, Szöllősi GJ, Ettema TJG, et al. [Phylogenomic analyses reveal that *Panguiarchaeu*m is a clade of genome-reduced Asgard archaea](https://www.biorxiv.org/content/10.1101/2025.02.13.637844v1). *bioRxiv.* doi:https://doi.org/10.1101/2025.02.13.637844.
9. Bergerat A, de Massy B, Gadelle D, Varoutas PC, Nicolas A, Forterre P. An atypical topoisomerase II from Archaea with implications for meiotic recombination. *Natur*e. 1997; 386:414-7.
10. Robert T, Vrielynck N, Mézard C, de Massy B, Grelon M. A new light on the meiotic DSB catalytic complex.. Semin *Cell Dev Biol*. 2016; 54:165-76.
11. Schlieper D, Oliva MA, Andreu JM, Löwe J. Structure of bacterial tubulin BtubA/B: Evidence for horizontal gene transfer. *Proc Natl Acad Sci U S*. 2005*;* 102: 9170–5.
12. Martin-Galiano AJ, Oliva MA, Sanz L, Bhattacharyya A, Serna M, Yebenes H, et al. Bacterial tubulin distinct loop sequences and primitive assembly properties support its origin from a eukaryotic tubulin ancestor. *J Biol Chem*. 2011; 286: 19789–803.
13. Jenkins C, Samudrala R, Anderson I, Hedlund BP, Petroni G, Michailova N, et al. Genes for the cytoskeletal protein tubulin in the bacterial genus Prosthecobacter. *Proc Natl Acad Sci U S A*. 2002; 99: 17049-54.
14. Rogers MB, Patron NJ, Keeling PJ. Horizontal transfer of a eukaryotic plastid-targeted protein gene to cyanobacteria. *BMC Biol*. 2007; 5:26. doi: 10.1186/1741-7007-5-26.
15. Guljamow A, Jenke-kodama H, Saumweber H, Quillardet P, Frangeul L, Castets AM, et al. Horizontal gene transfer of two cytoskeletal elements from a eukaryote to a cyanobacterium. *Curr Biol*. 2007; 17: 757–9.
16. Guljamow A, Delissen F, Baumann O, Thünemann AF, Dittmann E. Unique properties of eukaryote-type actin and profilin horizontally transferred to cyanobacteria. *Plos One*. 2012;7(1):e29926. doi: 10.1371/journal.pone.0029926.

**
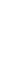

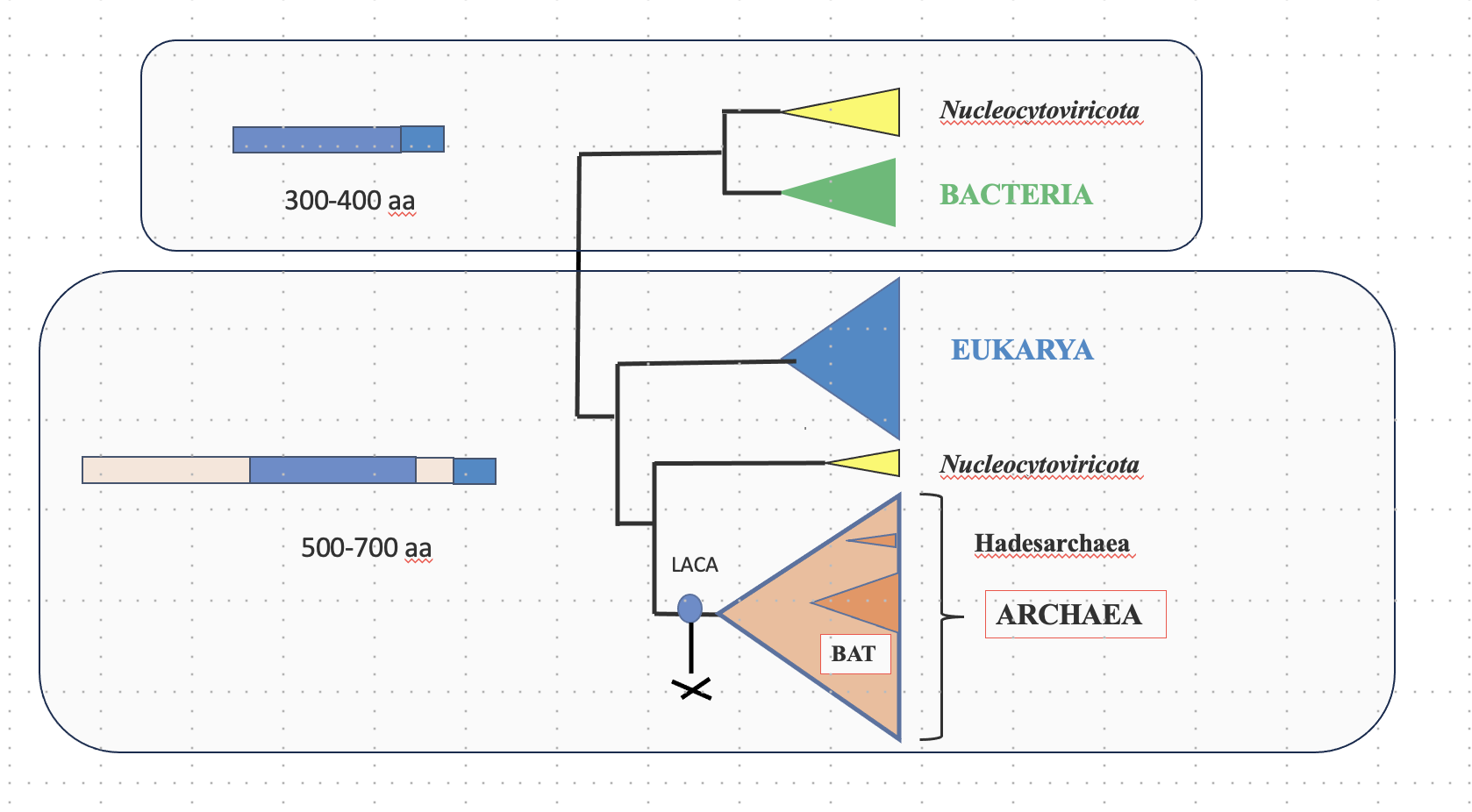
**

**Figure S1:** Schematic structure and tree of type IB DNA topoisomerases. Blue sections indicate homologous regions between the short and long forms (14). Adapted from published phylogenies (15, 17).


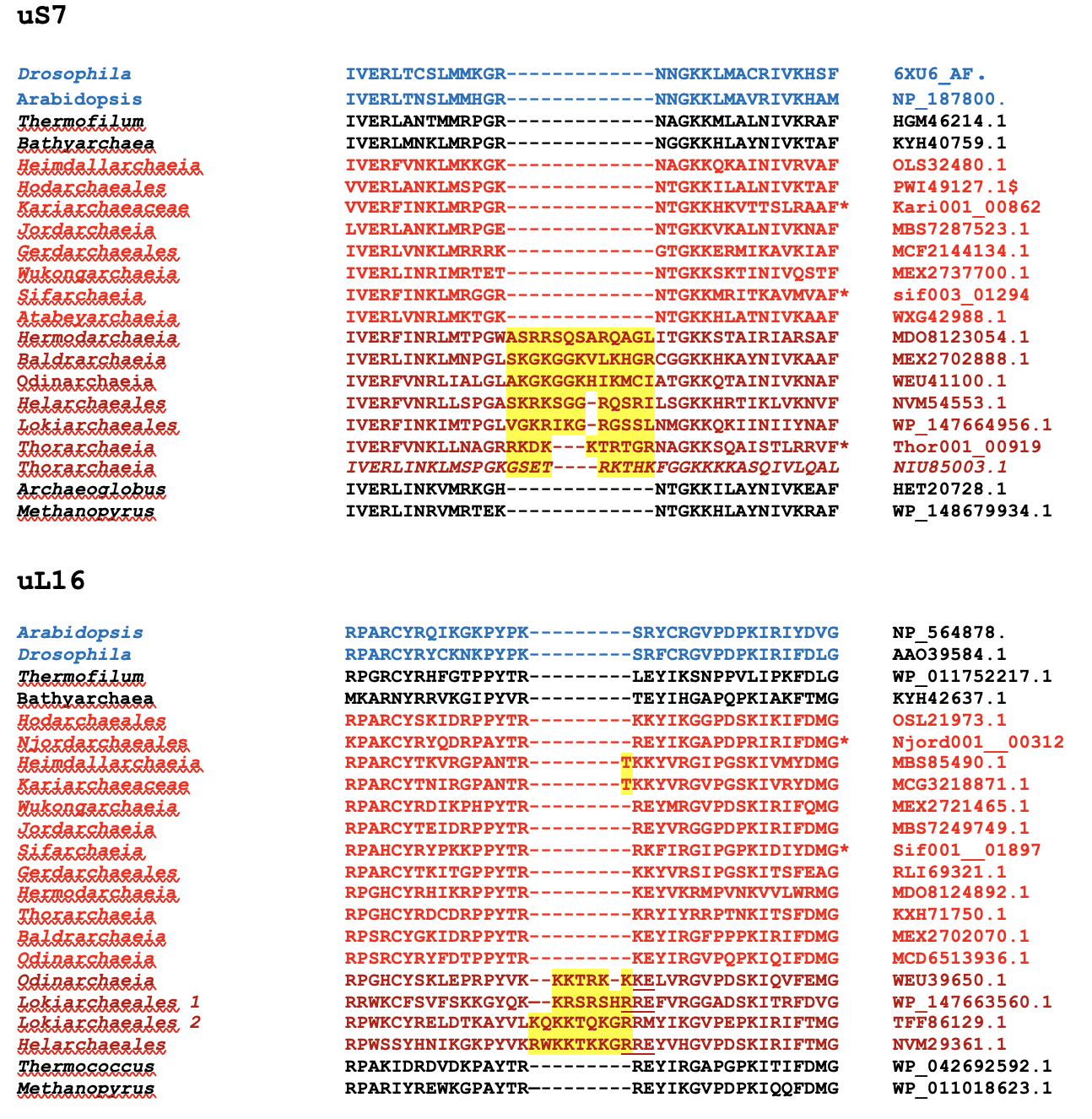


**Figure S2**: **Insertions present in the ribosomal proteins uS7 and uL16 in some lineages of Asgard archaea**. I have verified that these insertions are present in all members of each indicated lineages in screening the NCBI nr database and a database kindly provided by Prof Meng-Li and Dr Chengxiang Gu (sequences labelled with one star). This is the case, except for the uL16 insertion that is only present in a subset of MAGs annotated as *Odinarchaeia* (the figure shows two examples with or without the insertion). BLAST with sequences of the figure allows to recover homologues with their coordinates in the NCBI nr database and the localization of these sequences in the proteins. The names of archaeal lineages are underlined. Sequences from *Eukarya* are in blue, sequences from non-Asgard Archaea are in black, sequences from Asgard archaea without the insertion are in red and sequences from Asgard archaea with the insertion are in brown. Amino-acids corresponding to the insertions are in yellow boxes.

.
